# Detection for New Biomarkers of Tuberculosis Infection Activity Using Machine Learning Methods

**DOI:** 10.3390/diseases14020066

**Published:** 2026-02-11

**Authors:** Anna An. Starshinova, Adilya Sabirova, Olesya Koroteeva, Igor Kudryavtsev, Artem Rubinstein, Arthur Aquino, Andrey S. Trulioff, Ekaterina Belyaeva, Anastasia Kulpina, Raul A. Sharipov, Ravil K. Tukfatullin, Nikolay Y. Nikolenko, Anton Mikhalev, Andrey A. Savchenko, Alexandr Borisov, Dmitry Kudlay

**Affiliations:** 1 Department of Mathematics and Computer Science, Saint Petersburg State University, 199034 Saint Petersburg, Russia; 2 Department of Medicine, Almazov National Medical Research Center of the Ministry of Health of the Russian Federation, 197341 Saint Petersburg, Russia; 3 Medical Department, Bashkir State Medical University, 450000 Ufa, Russia; 4 Institute of Experimental Medicine, Acad. Pavlov St. 12, 197376 Saint Petersburg, Russia; 5 The Moscow Research and Clinical Center for Tuberculosis Control of the Moscow Government Department of Health, 127006 Moscow, Russia; nynikolenko@ya.ru; 6 Artificial Intelligence Center, Siberian Federal University, 660041 Krasnoyarsk, Russia; asmikhalev@yandex.ru; 7 Federal Research Center «Krasnoyarsk Science Center» of the Siberian Branch of the Russian Academy of Sciences, Scientific Research Institute of Medical Problems of the North, 660036 Krasnoyarsk, Russia; 8 Department of Pharmacology, Institute of Pharmacy, Sechenov University, 119002 Moscow, Russia; 9 NRC Institute of Immunology FMBA of Russia, 115522 Moscow, Russia; 10 Faculty of Bioengineering and Bioinformatics, Lomonosov Moscow State University, 119991 Moscow, Russia

**Keywords:** latent tuberculosis infection, preclinical stage, *Mycobacterium tuberculosis*, immune biomarkers, interferon signature, extracellular vesicles, transcriptomics, immunodiagnostics, PET/CT imaging, multidrug-resistant tuberculosis

## Abstract

Background/Objectives: Latent tuberculosis infection (LTBI) represents a critical reservoir for subsequent development of active tuberculosis (ATB) and poses significant challenges for early diagnosis and disease prevention. Traditional immunological assays, such as interferon-gamma release assays (IGRAs), are limited in their ability to reliably distinguish LTBI from ATB. Recent advances in high-throughput omics technologies and machine learning (ML) approaches offer new opportunities for precise, biomarker-based differential diagnostics. Methods: Transcriptomic and proteomic profiling of host immune responses has revealed reproducible gene and protein signatures associated with LTBI and ATB. The integration of ML techniques—including feature selection, dimensionality reduction, multimodal learning, and explainable AI—facilitates the construction of robust diagnostic models. Single-modality signatures, derived from RNA-seq, microarrays, or proteomic assays, are complemented by multimodal approaches that incorporate soluble mediators, immunological readouts, and imaging-derived features. Deep learning frameworks, such as convolutional neural networks and transformer-based architectures, enhance the extraction of complex molecular and structural patterns from high-dimensional datasets. Results: ML-driven analyses of transcriptomic and proteomic data consistently outperform conventional immunological tests in terms of sensitivity, specificity, and clinical applicability. Multimodal integration further improves diagnostic accuracy and robustness. These advances support the translational development of concise, quantitative reverse transcription PCR (qRT-PCR)-based biomarker panels suitable for routine clinical application, enabling early and reliable differentiation between LTBI and ATB. Overall, the combination of high-throughput omics and AI-based analytical frameworks provides a promising pathway for enhancing global tuberculosis diagnostics. Conclusions: This review provides a structured and critical synthesis of transcriptomic and proteomic biomarker research for LTBI and ATB discrimination, with a particular emphasis on machine learning–based analytical frameworks. Unlike previous narrative reviews, we systematically compare data-generating platforms, modelling strategies, validation approaches, and sources of heterogeneity across studies. We further identify key translational barriers, including cohort homogeneity, platform dependency, and limited external validation, and propose directions for future research aimed at improving clinical applicability.

## 1. Introduction

Tuberculosis (TB) remains a major global public health problem despite substantial progress in its control over recent decades. A particular challenge in contemporary phthisiology is latent tuberculosis infection (LTBI), which represents a large reservoir of TB and serves as a potential source for the future development of active TB. Accurate differentiation between LTBI and active TB is therefore essential for effective TB control, influencing epidemiological surveillance, preventive therapy, and the interruption of transmission.

The Russian Federation was removed from the list of high TB burden countries in 2022 [1]. According to the World Health Organization (WHO) Global Tuberculosis Report, the incidence of TB in Russia in 2023 was 38 cases per 100,000 population, reflecting a 43% reduction compared with 2015, while TB-related mortality declined by 58% over the same period [2]. These favourable trends indicate the effectiveness of national TB control measures; however, LTBI continues to pose a significant clinical and epidemiological challenge, particularly with regard to the timely identification of individuals at risk of progression to active disease [3,4].

Globally, TB incidence increased following the COVID-19 pandemic. In 2023, an estimated 10.8 million people developed TB worldwide, corresponding to 134 cases per 100,000 population. A steady rise in new cases was observed between 2021 and 2023 (10.4 million in 2021, 10.7 million in 2022, and 10.8 million in 2023) [2]. The pandemic disrupted TB diagnostic services and preventive programmes and is believed to have contributed to the reactivation of LTBI, particularly among individuals who did not receive preventive treatment, recent contacts of infectious cases, and immunocompromised patients [5].

The COVID-19 pandemic has also been associated with long-term alterations of immune function. Individuals with LTBI are characterised by an increased risk of progression to active TB under conditions of immune dysregulation, including post-COVID immune disturbances and post-COVID syndrome [6]. In this context, the ability to distinguish LTBI from active TB acquires particular importance, both for individual patient management and for public health decision-making.

Despite the overall decline in TB incidence and mortality in Russia, LTBI remains highly relevant, especially in the post-COVID era. This necessitates further refinement of early diagnostic approaches and risk stratification tools aimed at identifying individuals with a high probability of disease activation [6]. At present, there is no method for the direct detection of LTBI in humans; its diagnosis is indirect and relies on the assessment of host immune responses to *M. tuberculosis* antigens [7,8].

Cell-mediated immunity plays a central role in controlling *M. tuberculosis* infection. CD4^+^ T lymphocytes are essential for immune protection due to their capacity to secrete interferon-gamma (IFN-γ), while CD8^+^ T lymphocytes also contribute through IFN-γ production, activation of macrophages, destruction of infected cells, and direct cytolytic activity against intracellular mycobacteria. Antigen-specific CD8^+^ T cells have been detected in individuals with both LTBI and active TB. Notably, CD8^+^ T lymphocytes specific for early secretory antigenic target 6 (ESAT-6) and culture filtrate protein 10 (CFP-10) are more frequently observed in patients with active TB than in those with LTBI, which may reflect recent antigen exposure and higher mycobacterial activity [9,10].

In recent years, advances in artificial intelligence (AI) and bioinformatics have opened new perspectives for improving the diagnosis of LTBI and predicting progression to active TB [11,12]. The integration of AI-based and machine-learning approaches offers promising opportunities for the identification of novel biomarkers associated with TB activity. Such approaches may enable more precise differentiation between latent infection and active disease, thereby supporting the development of predictive, personalized TB prevention strategies. Building on these developments, the present review systematically examines transcriptomic and proteomic biomarker research aimed at distinguishing LTBI from active TB. We focus on the application of machine-learning methodologies, the comparison of data-generating platforms, and strategies for model validation. By highlighting sources of heterogeneity and translational barriers, this work identifies key challenges and proposes directions for future research to enhance clinical applicability and improve global TB control.

## 2. Machine Learning Technologies

Machine learning (ML) is a computational approach that employs algorithms and predictive models to automatically extract patterns from input data for the purpose of forecasting and decision-making. In contrast to traditional statistical techniques, ML methods are capable of efficiently processing large volumes of heterogeneous information and identifying latent structures, which renders them a valuable component of clinical decision support and personalized medicine. Within biomedical diagnostics, ML is applied to complex, high-dimensional datasets—including genomic, proteomic, and metabolomic profiles—facilitating the identification of subtle nonlinear patterns that may serve as novel biomarkers and improve the sensitivity and specificity of diagnostic systems. Against this background, the application of ML to the differential diagnosis of latent tuberculosis infection (LTBI) is of particular interest [13].

Machine learning has proven to be a powerful tool for tuberculosis (TB) research and clinical management. Its applications include diagnosing active tuberculosis (ATB), differentiating TB from non-tuberculosis mycobacterial (NTM) diseases, determining drug susceptibility, and supporting drug-discovery efforts [5]. However, its application to the differential diagnosis of LTBI remains relatively limited.

One of the major limitations in this field stems from the scarcity of suitable training data. The principal data sources used in machine learning—medical imaging, clinical information, and biomarker datasets—are restricted in LTBI. Patients with LTBI do not exhibit clinical symptoms or radiological abnormalities, making imaging and clinical data unsuitable for accurate diagnostic modelling. Consequently, ML-based differential diagnosis of LTBI must rely exclusively on biomarker data. At present, transcriptomic and proteomic biomarkers represent the most thoroughly investigated and promising sources for ML applications [14].

Given these constraints, this article focuses on recent advances in ML techniques that employ transcriptomic and proteomic data for the precise differential diagnosis of LTBI. Key challenges in this domain, as well as future technological directions, will also be discussed [11,12].

Recent progress in ML has opened new possibilities for distinguishing LTBI from ATB. Modern ML algorithms enable the accurate classification of LTBI and ATB using immunological, imaging-derived (where applicable), and molecular biomarkers. This review summarises the immunological mechanisms underlying LTBI, established diagnostic methods, and the most recent developments in ML applied to LTBI diagnostics. It also evaluates the principal methodological considerations, including feature selection, sample-size limitations, overfitting risks, model validation strategies, and the relative strengths and limitations of current ML techniques. While ML demonstrates considerable potential for improving diagnostic reliability, this requires rigorous data-preprocessing pipelines and high-quality analytical workflows. Ultimately, such efforts may substantially improve the diagnostic accuracy of pulmonary TB and strengthen clinical decision-making [14].

### 2.1. Pre-Analytical Quality Control and Data Integrity

A critical determinant of successful ML-based biomarker discovery is the stringent control of pre-analytical factors. The high sensitivity of omics technologies makes them highly vulnerable to variation during sample collection and processing. Strict standardisation of protocols for handling biological samples—such as whole blood, PBMCs, or serum—is essential, including precise documentation of pre-stabilisation intervals, centrifugation conditions, and freezing procedures. Antigen-dependent protocols, particularly those involving PPD or ESAT-6/CFP-10 stimulation, require special attention due to their substantial influence on reproducibility across study centres [15,16].

Quality control procedures span all technological platforms:

- assessment of read quality and adapter trimming in RNA-seq;
- amplification-efficiency checks in qRT-PCR;
- hybridisation validation for microarray datasets;
- chromatographic-peak verification in proteomics.

### 2.2. Normalization and Correction of Technical Variability

Normalization and correction for technical variation represent another essential stage. Omics platforms are highly susceptible to batch effects arising from differences in experimental conditions, reagent lots, or processing times. Failure to correct for such artefacts may lead ML models to learn technical rather than biological signals. Platform-specific normalisation techniques include: RMA for microarray data; DESeq2 and TMM for RNA-seq; Ct-based expression quantification for qRT-PCR.

Residual technical variation is typically investigated using dimensionality-reduction approaches such as PCA [17] and UMAP [18], while correction is frequently performed with ComBat, an empirical Bayes method [19]. For single-cell RNA-seq, advanced integration frameworks—such as Harmony [20] and Scanorama [21]—allow the harmonisation of multi-batch datasets without loss of biological signal.

### 2.3. Feature Selection and Model Development

Feature selection and model construction follow the principle of minimising overfitting while retaining maximal biological informativeness. A two-step strategy is typically applied:

1. Initial screening of differentially expressed genes with false-discovery-rate control to generate a preliminary feature set.
2. Robust feature selection using regularisation techniques and ensemble methods.

Validation of feature stability is performed using bootstrap analysis and stratified cross-validation.

Model development seeks an optimal balance between performance and interpretability. Consequently, interpretable algorithms—regularised logistic regression, support vector machines, ensemble methods, and neural networks with explainability layers—are often preferred. Validation strategies incorporate independent test sets, external validation cohorts, and comprehensive metrics assessing both discrimination (AUC-ROC, sensitivity, specificity) and probability calibration.

### 2.4. Biological Interpretation and Explainability

The final stage involves biological interpretation and explainability. Biological validation includes analyses of gene ontology enrichment, molecular pathways, and protein–protein interaction networks, ensuring consistency of the identified gene signatures with known TB pathogenesis.

To enhance clinical transparency, explainable-AI tools such as SHAP (SHapley Additive exPlanations) [22] and LIME (Local Interpretable Model-agnostic Explanations) [23] are widely employed. Feature-importance analyses further support interpretation at both global and individual levels. Visualisation tools—including expression heatmaps, feature-importance rankings, and dependency plots—facilitate comprehension by clinicians.

### 2.5. Reproducibility and Reporting Standards

Ensuring full reproducibility is essential for the successful clinical translation of ML-based diagnostic methods.

This is achieved through:

- publication of analysis code with containerisation;
- comprehensive documentation of software versions and analytical parameters;
- adherence to international guidelines (e.g., TRIPOD, MIAME);
- registration of study protocols in open-access repositories.

Such measures ensure not only robust statistical outcomes but also reliable integration into clinical practice, thereby supporting the development of personalised diagnostic and prognostic tools for tuberculosis.

Although ML is widely used in diagnosing active TB, differentiating NTM diseases, predicting drug resistance, and supporting drug discovery, its application to the differential diagnosis of LTBI remains uncommon due to limited data availability. Because LTBI lacks clinical and radiological features, ML approaches must rely entirely on biomarker data. Currently, transcriptomic and proteomic biomarkers are the most extensively studied and promising for ML-based LTBI diagnostics. Based on these considerations, the present review outlines the conceptual foundations of ML, summarises commonly used algorithms, and focuses on ML approaches using transcriptomic and proteomic data for the precise diagnosis of latent tuberculosis infection [12].

## 3. Transcriptomics

Transcriptomic approaches occupy a central position among biomarkers of *Mycobacterium tuberculosis* (MTB) infection activity, as they capture the host’s integrated cellular response to pathogen exposure. Alterations in the expression of both coding and non-coding transcripts emerge across different stages of infection and reflect dynamic host–pathogen interactions. These changes are detected using two principal whole-transcriptome profiling platforms: microarray-based hybridisation techniques and RNA sequencing (RNA-seq). While microarrays rely on the hybridisation of pre-labelled probes with complementary cDNA targets, RNA-seq directly sequences cDNA fragments using next-generation sequencing technologies, providing a more comprehensive and quantitative characterisation of transcript abundances.

With the rapid development of machine learning (ML) and artificial intelligence (AI), integrative analyses combining diverse transcriptomic datasets and ML frameworks have become feasible, enabling improved discrimination of latent tuberculosis infection (LTBI) and active tuberculosis (ATB) [11,12]. Continuous advances in AI-driven analytical frameworks now allow multidimensional integration of transcriptomic signatures, creating new opportunities for LTBI–ATB discrimination and for predicting progression from latent to active disease.

Host cells infected with MTB mount pathogen-specific immune responses, resulting in differential expression of coding and non-coding transcripts across immune and tissue-resident cell populations. Transcriptomics therefore remains a key tool for elucidating these cellular processes. Furthermore, emerging evidence suggests that metabolic transcript signatures and cell-type-specific gene expression patterns may provide additional diagnostic value beyond conventional immune response genes. The integration of cell-specific and whole-blood transcriptomic profiles through ML has also been used to identify subsets of LTBI individuals with an expression profile more similar to active disease, potentially indicating higher progression risk.

A growing body of research has applied ML-based transcriptomic methods specifically to the differential diagnosis of LTBI and ATB. In addition to the canonical signatures, such as those summarised in Table 1, recent studies have reported alternative gene panels and integrative models demonstrating robust performance across independent cohorts. Notably, bioinformatics analyses have identified high-performing signatures, including metabolic DEGs that distinguish LTBI from ATB with AUC values approaching ~0.87 in cross-cohort validations [10], and multi-cohort expression analyses have produced three-gene immune-related signatures (e.g., SLC26A8, ANKRD22, FCGR1B) capable of discriminating LTBI from ATB (AUC~0.80).

Additionally, comparative studies between transcriptomic signatures and established immunodiagnostics (e.g., IGRA) among household contacts in high-incidence populations support the utility of small gene panels (e.g., FCGR1B, GBP1, GBP5) for rapid LTBI screening. These recent findings highlight both the diversity of transcriptomic approaches and the ongoing need for validation across larger and geographically diverse cohorts.

The table summarises major transcriptomic signatures developed to differentiate latent tuberculosis infection (LTBI) from active tuberculosis (ATB). It includes the data-generating platforms, gene composition of each signature, diagnostic performance metrics (AUC, sensitivity, specificity), and the clinical context in which each signature was evaluated. The table also highlights signatures that have progressed to translational validation using qRT-PCR and those demonstrating potential suitability for point-of-care formats

### Critical Appaisal of Transcriptomic Approaches in LTBI and ATB Differentiation

While transcriptomic profiling offers unprecedented insights into host responses to *Mycobacterium tuberculosis* (MTB) infection, several limitations must be acknowledged, particularly in the context of translating these findings into robust clinical diagnostics.

Many transcriptomic studies rely on extremely small discovery cohorts. For instance, some RNA-seq and microarray studies have included as few as 4–10 participants per group Lu et al. [26] Table 1. Such limited sample sizes compromise statistical power, increase the risk of false discoveries, and reduce confidence in the generalisability of identified gene signatures.

A significant proportion of published transcriptomic studies have been conducted in single geographic regions, predominantly in China, with limited inclusion of ethnically and genetically diverse populations. Consequently, the applicability of many signatures to global populations remains uncertain, and validation across multi-ethnic cohorts is necessary to ensure robust performance [28].

Studies differ in the choice of sample type (whole blood vs. isolated PBMCs) and stimulatory conditions (e.g., PPD vs. ESAT-6/CFP-10). Such variability produces inconsistent immunological and transcriptomic signatures, making it difficult to establish a standardised diagnostic panel applicable across laboratories and study sites.

Most studies employ cross-sectional designs, which capture a snapshot of transcrip-tional changes at a single time point. While useful for differentiating LTBI from ATB, these designs do not provide longitudinal insights into disease progression, limiting the ability to predict which individuals with LTBI will develop active TB.

High-performing gene signatures often lack rigorous validation in independent cohorts. For example, classifiers based on BATF2, UBE2L6, or multi-gene immune panels have demonstrated excellent performance in discovery datasets but are sometimes untested in external populations. This raises concerns about overfitting and dataset-specific performance [12].

Complex machine-learning models, especially those employing deep learning or integrative multi-omics, frequently suffer from limited interpretability. Clinicians may find it difficult to trust models whose decision-making processes are opaque, potentially hindering adoption in clinical practice. Incorporating interpretable ML frameworks and providing clear biological rationale for selected features are therefore essential for translation.

The analytical platform used (e.g., microarray vs. RNA-seq) and batch effects across experimental runs can substantially influence transcriptomic measurements. Differences in preprocessing, normalization, and sequencing protocols may result in variability in signature performance across studies, limiting reproducibility and clinical transferability.

Many transcriptomic signatures involve large gene panels, whole-transcriptome profiling, or qRT-PCR validation of multiple targets. The high cost, need for specialised equipment, and technical expertise required can limit applicability in resource-constrained, high TB-burden settings, where rapid and affordable diagnostics are most needed.

Despite these limitations, recent developments in AI-driven integrative analyses, single-cell transcriptomics, and cross-cohort validations show promise. Multi-cohort three-gene signatures (e.g., SLC26A8, ANKRD22, FCGR1B) and metabolic transcript markers have demonstrated moderate-to-high diagnostic accuracy and improved generalisability. Further validation and simplification of such panels could enhance their clinical utility, particularly for point-of-care applications [20,28].

## 4. Microarray-Based Approaches

ML methods applied to microarray datasets offer a promising strategy for accurately distinguishing ATB from LTBI. In one prospective cohort study, differential gene expression analysis using microarrays, combined with quantitative reverse-transcription PCR (qRT-PCR), identified four candidate biomarkers relevant for LTBI–ATB discrimination: nuclear export mediator, spermatogenesis regulator, DEAH-box polypeptide 29, and protein tyrosine phosphatase receptor type C (PTPRC). A naïve Bayes classifier constructed using spermatogenesis regulator, DEAH-box protein 29, and PTPRC achieved high sensitivity (97.9%) and accuracy (97.8%). However, the model lacked external validation, limiting generalizability.

To address this limitation, Lu et al. conducted an independent study analysing microarray-derived gene expression profiles in three cohorts: ATB, LTBI, and healthy controls (HCs). They identified three discriminatory genes—chemokine (C-X-C motif) ligand 10 (CXCL10), ATP-binding cassette phospholipid transporter ATP10A, and Toll-like receptor 6 (TLR6)—and developed a diagnostic model using a decision tree algorithm. In an external validation cohort (*n* = 42 ATB, *n* = 55 LTBI, *n* = 22 HCs), the model achieved a sensitivity of 71% and specificity of 89% [26].

Importantly, the discovery cohort included only four participants per group, which may substantially reduce model reliability. Adequate cohort size is essential for statistical power, generalizability, and robustness of ML-based diagnostic tools. Larger, more diverse datasets improve the model’s ability to capture representative transcriptional patterns and reduce the risk of overfitting. They also allow more precise estimation of performance metrics, narrower confidence intervals, and reliable subgroup analyses—factors crucial for assessing clinical applicability across heterogeneous populations. Future efforts to develop transcriptomic-based LTBI diagnostic models should incorporate rigorous sample size estimation and ensure validation across independent, demographically diverse cohorts.

## 5. RNA Sequencing

RNA sequencing (RNA-seq) offers several advantages over microarray-based approaches, including higher throughput, superior sensitivity, and the ability to detect novel transcripts and genetic variants. Using RNA-seq, researchers have successfully identified biomarkers capable of distinguishing active tuberculosis (ATB) from latent tuberculosis infection (LTBI), employing advanced machine learning (ML) algorithms for differential gene analysis and classification [27].

In a study by Wang et al., RNA-seq was combined with unsupervised clustering of gene expression profiles derived from PPD-stimulated samples across three groups—ATB, LTBI, and healthy controls (HCs) [28]. The analysis identified a three-gene signature consisting of TNFRSF10C (tumour necrosis factor receptor superfamily member 10C), EBF3 (early B-cell factor 3), and A2ML1 (alpha-2-macroglobulin-like 1). This signature achieved a classification accuracy of 91.5%, with a sensitivity of 86.2% and specificity of 94.9% for distinguishing the groups. To evaluate its diagnostic potential, the authors further tested the three-gene panel in a cohort of 147 individuals with suspected ATB. The model achieved 82.4% sensitivity and 92.4% specificity in identifying active disease. These findings demonstrate that combining multiple transcriptomic biomarkers with ML algorithms can substantially enhance diagnostic accuracy for LTBI.

However, caution is warranted when interpreting these results. All referenced RNA-seq and microarray studies were conducted in a single geographical region (China), which may limit the generalisability of derived models to genetically diverse populations. Additionally, the relatively small sample sizes—particularly in the microarray-based investigations—underscore the need for validation in larger, multi-centre cohorts to ensure robustness and external validity.

**Figure 1:**
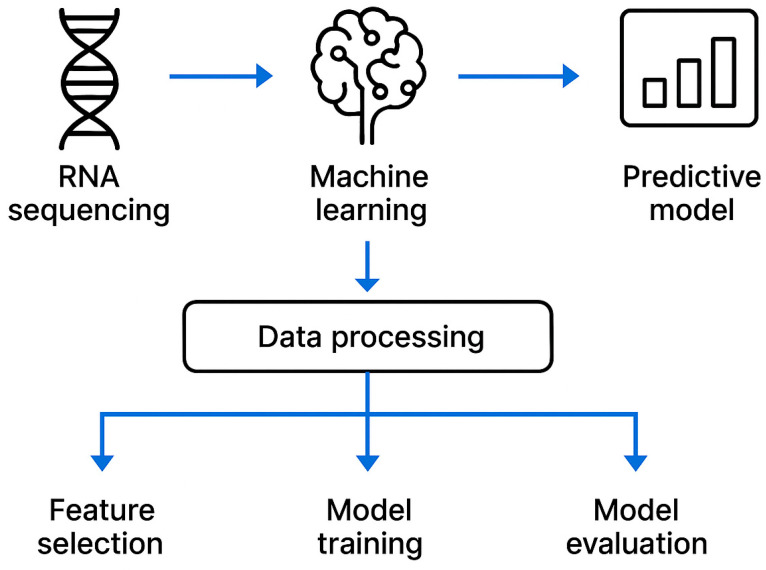
ML-driven RNA-sequencing process.

Overall, ML-driven RNA-seq biomarker discovery holds considerable promise for clinical application. Nevertheless, population-specific variation in biomarker performance remains a challenge, and further research is necessary to confirm the clinical utility of identified transcriptomic signatures across global settings (Figure 1).

### 5.1. Real-Time Polymerase Chain Reaction (RT-PCR)

**Figure 2:**
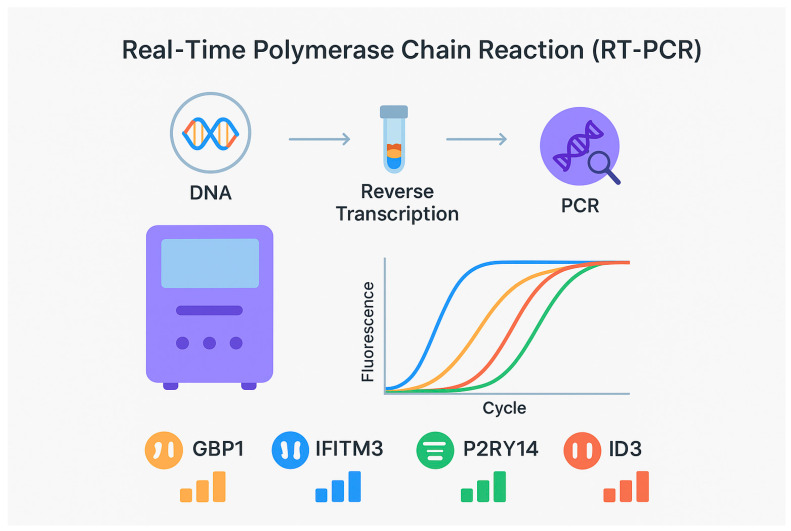
PCR (RT-PCR) combined with ML approaches.

Developing LTBI diagnostic models using microarray or RNA-seq technologies can be challenging due to their cost, complexity, and limited feasibility in resource-constrained settings. Recent studies, however, demonstrate that real-time PCR (RT-PCR) combined with ML approaches offers an affordable and practical alternative for improving LTBI diagnostics (Figure 2).

A landmark study from Bangalore, India, utilised RT-PCR and ML-based modelling of gene expression data to identify a concise diagnostic signature [24]. This work yielded a four-gene combination comprising GBP1 (guanylate-binding protein 1), IFITM3 (interferon-induced transmembrane protein 3), P2RY14 (UDP-glucose-specific G(i)-coupled P2Y receptor), and ID3 (inhibitor of DNA binding 3).

This signature demonstrated robust performance in external validation cohorts:

- Gambia: AUC 0.89; sensitivity 85%; specificity 76%
- Uganda: AUC 0.82; sensitivity 73%; specificity 78%

The study is notable for its relatively large and diverse participant pool spanning ATB, LTBI, and HC groups, which strengthened the reliability of biomarker identification and model validation. Its successful cross-continental validation also highlights the potential applicability of RT-PCR–based ML diagnostics across varied epidemiological settings.

Importantly, not all transcriptomic investigations require ML integration to achieve high diagnostic accuracy. Several studies have demonstrated strong results using transcriptomic analysis alone.

A prospective case–control study identified a six-gene whole-blood signature—GBP2, FCGR1B, SERPINC1, TUBB6, TRMT2A, and SDR39U1—which achieved an AUC of 0.930, with 90.9% sensitivity and 88.5% specificity for distinguishing ATB from LTBI [12]. Another study combined differential gene expression profiling, co-expression network analysis, and short time-series analysis of public GEO datasets, identifying four biomarkers: UBE2L6, BATF2, SERPING1, and VAMP5. Their combined performance—88% sensitivity and 78% specificity for ATB—substantially exceeded that of the T-SPOT assay (75.3% sensitivity; 69.1% specificity).

Collectively, these examples highlight the diagnostic potential of transcriptomic signatures—whether ML-enhanced or not—for differentiating LTBI from ATB and underscore their promise for improving global TB diagnostics.

Developing diagnostic models for LTBI using microarray or RNA-seq technologies can be challenging due to their complexity and high cost, which limits their implementation in resource-constrained settings. However, recent studies have demonstrated that RT-PCR combined with machine learning (ML) algorithms may offer an accessible and user-friendly alternative for improving LTBI diagnostics. In a study conducted in Bangalore, India, RT-PCR and ML algorithms were applied to model gene expression data [26]. This approach resulted in the development of a four-gene signature comprising guanylate-binding protein 1 (GBP1), interferon-induced transmembrane protein 3 (IFITM3), the G(i)-protein-coupled and UDP-glucose-specific P2Y receptor (P2RY14), and inhibitor of DNA binding 3 (ID3), which demonstrated promising diagnostic performance. In a validation cohort from The Gambia, the four-gene combination yielded an area under the curve (AUC) of 0.89, with a sensitivity of 85% and a specificity of 76%. In a Ugandan cohort, the AUC was 0.82, with a sensitivity of 73% and a specificity of 78%. This study is notable for its relatively large number of participants with active TB (ATB), LTBI, and controls, thereby providing robust identification and validation of potential biomarkers. In addition, the study demonstrated high sensitivity, specificity, and diagnostic accuracy of the LTBI classification model across different national cohorts, highlighting its potential applicability in diverse geographical settings.

Importantly, not all transcriptomic studies require the integration of machine-learning algorithms to achieve high diagnostic performance for LTBI. Some investigations have reported robust outcomes using transcriptomic analysis alone to identify potential biomarkers suitable for LTBI differential diagnosis. In a prospective case–control study employing whole-blood transcriptomics, a six-gene signature—consisting of guanylate-binding protein 2, Fc gamma receptor 1B, serpin peptidase inhibitor clade C member 1, gamma-tubulin-associated protein 6, tRNA methyltransferase 2 homolog A, and short-chain dehydrogenase/reductase family 39U member 1—was identified and evaluated. This six-gene combination achieved an impressive AUC of 0.930, with 90.9% sensitivity and 88.5% specificity for distinguishing ATB from LTBI [29].

In another study, researchers integrated differentially expressed genes, co-expression network analysis, and short-time series analysis. Using transcriptomic datasets from the Gene Expression Omnibus, they identified four biomarkers (UBE2L6, BATF2, SERPING1, and VAMP5). The combined biomarker panel achieved a diagnostic sensitivity of 88% and specificity of 78% for ATB, significantly outperforming T-SPOT, which demonstrated a sensitivity of 75.3% and a specificity of 69.1%. These examples underscore the diagnostic potential of transcriptomic analysis alone for biomarker discovery and the differential diagnosis of LTBI, enabling high diagnostic accuracy without additional computational layers.

### 5.2. Single-Cell RNA Sequencing (scRNA-Seq)

scRNA-seq is a powerful analytical tool that enables detailed investigation of cellular heterogeneity and gene-expression profiles at the single-cell level. Although scRNA-seq is primarily used to study gene-expression patterns and cellular dynamics in various diseases, it also offers potential utility in elucidating the biology and improving the diagnosis of tuberculosis. By applying scRNA-seq, researchers can examine transcriptomic characteristics of individual immune cells in patients with LTBI, obtaining detailed insights into cellular composition, functional states, and immune responses associated with latent infection. Below are potential areas in which scRNA-seq may contribute to LTBI research and diagnostics:

(1) *Identification of LTBI-specific gene-expression features.*

scRNA-seq enables the discovery of gene-expression patterns and molecular signatures characteristic of LTBI. By comparing the single-cell transcriptional profiles of immune cells from LTBI individuals with those from uninfected or ATB subjects, differentially expressed genes or gene modules indicative of LTBI may be identified.

(2) *Characterisation of immune-cell subpopulations.*

scRNA-seq facilitates the identification and detailed characterisation of immune-cell subsets associated with LTBI. Single-cell transcriptomic analysis can reveal distinct immune-cell clusters that emerge during LTBI, including T cells, B cells, macrophages, dendritic cells, and others. This enables a deeper understanding of cellular dynamics, interactions, and functional states during latent infection.

(3) *Assessment of immune-cell activation and response.*

scRNA-seq can reveal activation states and functional responses of immune cells during LTBI. By examining single-cell gene-expression profiles, researchers can identify specific cell subsets and pathways involved in the host immune response to *Mycobacterium tuberculosis* (MTB). Such information helps clarify immune mechanisms underlying LTBI and may support the identification of diagnostic or therapeutic targets.

It is important to note that the use of scRNA-seq for LTBI diagnosis remains in its early stages, and further studies are required to fully realise its potential. ML models based on scRNA-seq for LTBI classification also require additional development and validation (Table 2).

The principal analytical applications of single-cell RNA sequencing (scRNA-seq) in the context of LTBI research. It categorises the role of scRNA-seq in identifying LTBI-associated genes, characterising immune-cell subpopulations, assessing activation of interferon-dependent pathways, and generating predictive machine-learning models based on high-resolution immune profiling. These applications provide mechanistic insight and support the development of risk-stratification tools.

External validation is typically performed using independent cohorts to assess model generalisability. Translational validation often involves qRT-PCR assays, with primer efficiency testing and ΔCt normalisation against reference genes to ensure reproducibility across platforms.

Single-cell RNA sequencing (scRNA-seq) enables unbiased, high-resolution profiling of gene-expression patterns at the level of individual cells, thereby capturing cellular heterogeneity that is obscured in bulk transcriptomic analyses. In the context of tuberculosis, scRNA-seq allows detailed characterisation of immune-cell subsets, activation states, and transcriptional programmes associated with latent infection. By comparing immune-cell landscapes between LTBI, ATB, and uninfected individuals, scRNA-seq facilitates the identification of LTBI-specific cellular phenotypes, interferon-driven transcriptional states, and immune trajectories linked to progression risk. These high-dimensional datasets further provide a foundation for machine-learning–based classification models capable of identifying high-risk LTBI molecular subtypes prior to clinical disease manifestation.

## 6. Proteomic Approaches for LTBI vs. Active TB

Proteomic approaches, combined with machine learning (ML) algorithms, have demonstrated considerable potential for identifying diverse biomarker candidates capable of differentiating latent tuberculosis infection (LTBI) from active tuberculosis (ATB). Research efforts have primarily focused on the diagnostic utility of Mycobacterium tuberculosis (MTB)-specific proteins, host-derived antibodies, and serum or plasma protein signatures.

### MTB-Specific Proteins

Selecting antigenic targets from approximately 4000 MTB-encoded proteins for accurate detection of latent infection presents a substantial challenge. As previously discussed, the tuberculin skin test utilises purified protein derivative (PPD), which does not discriminate reliably between prior BCG vaccination and environmental non-tuberculous mycobacteria, resulting in reduced specificity [10].

High-throughput antibody profiling studies further demonstrated that serological responses to MTB antigen panels (e.g., Rv0934, Rv1860, Rv1827, Rv3881c) differentiate ATB from LTBI, with moderate sensitivity and high specificity, supporting the complementary role of host antibody signatures [turn0search9, turn0search10]. Additionally, affinity proteomics using proximity extension assays identified inflammatory and cytokine-related proteins (IFN-γ, LIF, uPA, CSF-1, SCF, SIRT2, 4E-BP1, GDNF) that discriminated active PTB from other respiratory conditions with AUCs exceeding 0.90, underscoring the potential of pathway-informed biomarker selection [28,29,30].

Overall, proteomic approaches, particularly when integrated with ML algorithms, provide a rich source of candidate biomarkers for LTBI vs. ATB discrimination. Future research should prioritise external validation, standardisation of platforms, and cost-effective assay development to translate discovery findings into clinically applicable diagnostic tools [31].

Although interferon-gamma release assays (IGRAs) improved diagnostic accuracy through the use of RD1-encoded antigens ESAT-6 (Rv3875) and CFP-10 (Rv3874)—effectively mitigating cross-reactivity with BCG and most environmental mycobacteria—they nonetheless cannot distinguish LTBI from ATB. Consequently, development of new LTBI discrimination tools must prioritise antigens associated with RD-LTBI (Table 3).

This table provides an overview of key proteomic biomarker classes investigated for LTBI and ATB discrimination, the analytical technologies used for their quantification, and associated methodological advantages and limitations. It further summarises the ways in which machine-learning algorithms have been applied to enhance biomarker selection, multiplex signature construction, and integration with transcriptomic data.

In a cohort study involving 440 patients, microarray analysis, clustering, and protein–protein interaction networks identified four biomarkers (Rv0934, Rv1827, Rv1860, and Rv3881c) with discriminatory potential [32]. These markers achieved 67.3% sensitivity and 91.2% specificity in differentiating ATB from LTBI, while ELISA validation yielded a sensitivity of 71.22% and specificity of 91.87%.

In a subsequent investigation, Rv0934 and Rv1827 were substituted with Rv2031c and Rv3803c, and a random forest model was applied to evaluate combinations of Rv1860, Rv3881c, Rv2031c, and Rv3803c [33]. This approach markedly improved sensitivity (93.3%) and specificity (97.7%) in both training and validation cohorts.

Both studies employed discovery and validation cohorts consisting exclusively of Chinese participants with comparable sample sizes; however, diagnostic performance varied substantially. This discrepancy likely reflects differences in antigen selection and ML algorithm choice. Li et al. (2021) selected Rv0934, Rv1827, Rv1860, and Rv3881c, whereas Li et al. (2022) selected Rv1860, Rv3881c, Rv2031c, and Rv3803c [10,32]. Assuming no substantial effect from algorithmic variation, inclusion of Rv2031c and Rv3803c may enhance model performance. Nonetheless, ML algorithms themselves can considerably affect diagnostic discrimination, further complicating optimization [32].

Unlike the above studies, which relied on four antigens and a single ML algorithm, Cao et al. expanded the MTB antigen pool to seven proteins (Rv1408, R0248, Rv2026c, Rv2716, Rv2031c, Rv2928, Rv2121c) and applied logistic regression and hierarchical clustering as ML strategies [33]. The combined seven-antigen signature yielded exceptional discrimination between cutaneous TB and tuberculous lymphadenitis, achieving an AUC of 0.9844, sensitivity of 96.77%, and specificity of 93.75% in the discovery set, with similarly robust performance in validation (AUC 0.9632; sensitivity 93.33%; specificity 93.10%).

Many studies rely on relatively small cohorts, often limited to a single geographic region or ethnic group (predominantly Chinese populations). This raises concerns about the generalisability of proteomic signatures to populations with different genetic backgrounds, environmental exposures, or comorbidities.

Diagnostic performance can vary substantially depending on the proteomic platform used (e.g., ELISA, Luminex, mass spectrometry, proximity extension assays). Such platform-dependent batch effects complicate cross-study comparisons and hinder standardisation for clinical application. Although internal validation is common, few signatures have been rigorously tested in independent, multi-centre, or multi-ethnic cohorts. Without robust external validation, the reported high sensitivities and specificities may overestimate performance in real-world settings.

The choice of MTB antigens or host-derived proteins can substantially influence model performance. Differences in antigen panels, assay conditions, or ML feature selection algorithms may lead to inconsistent results across studies, as observed in variations between Rv0934/Rv1827 and Rv2031c/Rv3803c-based models.

Many proteomic signatures rely on multiplex panels, advanced analytical platforms, or multi-omics integration. Such approaches are often expensive, technically demanding, and time-consuming, limiting their applicability in high-burden, resource-limited settings where LTBI and ATB diagnosis is most critical.

Proteomic signatures reflect the host’s immune and inflammatory responses, which may fluctuate over time due to infection stage, comorbidities, or recent vaccination. This temporal variability can reduce reproducibility and complicate the establishment of universal diagnostic thresholds.

While multi-omics approaches (e.g., transcriptomics + proteomics + lipidomics) hold promise for improving accuracy, integrating heterogeneous data layers remains technically challenging. Computational complexity and potential overfitting may limit clinical translation unless models are rigorously validated.

In summary, while proteomics combined with ML provides a rich source of candidate biomarkers for LTBI vs. ATB discrimination, these limitations highlight the need for large-scale, multi-ethnic cohort validation, standardised measurement protocols, simplified multiplex panels, and integration with complementary omics and clinical data before routine clinical application.

## 7. Host-Specific Antibodies and Cytokines

In addition to MTB-derived antigens, host-specific antibodies and cytokines show considerable promise as diagnostic biomarkers for tuberculosis. Several studies have explored the discriminatory utility of such host immune mediators for separating ATB from LTBI.

A proteomic microarray analysis identified a panel of 15 MTB antigen-specific antibodies capable of distinguishing ATB from LTBI with high sensitivity (85.4%) and specificity (90.3%) [34]. However, the panel has not undergone validation using alternative technologies, and its size may limit both cost-effectiveness and feasibility in clinical practice.

Another strategy utilised logistic regression to derive a cytokine signature comprising CCL1, CXCL10 (IP-10), vascular endothelial growth factor (VEGF), and adenosine deaminase 2 (ADA2), achieving 95% sensitivity and 90% specificity in distinguishing ATB from LTBI [35]. Specificity varied across control groups with differing demographic and clinical backgrounds, underscoring the need for multicentre validation prior to clinical adoption.

Multi-protein biosignatures have also shown diagnostic utility. A study conducted in sub-Saharan Africa identified a panel of nine serum proteins with 92% sensitivity and 71% specificity [36]. In another landmark study, proteomic fingerprinting combined with support vector machine (SVM) classification yielded a four-protein signature achieving 94% diagnostic accuracy independent of HIV status [37].

In China, a model incorporating antigen-specific biomarkers (ESAT-6, CFP-10), IFN-γ, erythrocyte sedimentation rate (ESR), and high-sensitivity C-reactive protein (hs-CRP) using random forests and bagging achieved sensitivity and specificity of 92.80% and 89.86%, respectively [38].

Similarly, integration of T-SPOT.TB results with plasma cytokine profiling identified a three-marker signature (including MCP-1) with AUC 0.94, sensitivity 87.76%, and specificity 91.84% for differentiating MTB infection stages [39].

Published studies illustrate various strategies for constructing sets of biomarkers. The serological approach [34], utilizing microarray-based proteome analysis of MTB, enabled the formation of a combination of 15 antigen-specific antibodies that provided high discrimination indices between active TB (ATB) and latent tuberculosis infection (LTI) with sensitivity (85.4%) and specificity (90.3%). Despite its informative nature, such an extensive panel requires validation through alternative technologies and may be limited in applicability due to cost and complexity.

A more targeted cytokine profile [35] derived via logistic regression included CCL1, CXCL10 (IP-10), VEGF, and ADA2 activity, achieving sensitivity of 95% and specificity of 90% for differentiation between ATB and LTI within the study cohort. However, specificity varied across control groups of varying composition, underscoring the need for multicenter validation considering age, comorbidity, and geography.

In Sub-Saharan African countries, a multiprotein signature composed of nine serum proteins achieved sensitivity of 92% and specificity of 71%, illustrating how addition of several immune mediators increases sensitivity but necessitates fine-tuning thresholds to optimize specificity [40]. More flexible ML algorithms often enhance discrimination power. For instance, proteomic ‘fingerprinting’ of serum using support vector machines [(SVM)] yielded a four-protein combination providing accuracy of approximately 94% irrespective of HIV status, demonstrating robustness against this important clinical confounder [41].

In a Chinese cohort, integration of antigen-specific markers (ESAT-6, CFP-10), interferon-gamma (IFN-γ), and routine inflammatory measures (ESR, hs-CRP) into a random forest model allowed distinction between ATB and LTI with sensitivity of 92.8% and specificity of 89.86%, highlighting practical utility of combining immunological and clinico-laboratory variables [38]. Using results from T-SPOT.TB as a starting point for selecting plasma cytokines led to a three-factor signature (including MCP-1, etc.) with area under curve (AUC) of 0.94, sensitivity of 87.76%, and specificity of 91.84% for differentiating stages of MTB infection [39].

Collectively, these examples demonstrate that multi-parameter signatures constructed using ML methods consistently outperform single markers and frequently surpass standard Interferon Gamma Release Assays (IGRA) regarding balance of sensitivity/specificity when addressing LTI vs. ATB challenges [42].

From a biological signaling perspective, the most reproducible signals include interferon-inducible chemokines (especially CXCL10/IP-10, occasionally CXCL9), innate response and vascular activation markers (such as VEGF), and enzymatic activities reflecting cellular immunity (ADA2). Notably, these soluble axes align with transcriptomic signatures where IFN-dependent genes (including CXCL10-associated modules) have repeatedly entered minimal signatures for differentiating ATB from LTI (for example, CXCL10/ATP10A/TLR6). This cross-platform convergence enhances plausibility of traits and transferability among centers and populations [43].

These studies highlight the potential use of diverse combinations of cytokine proteins and ML algorithms for detecting LTBI [44]. Such approaches are promising both operationally efficient and economically viable as discriminatory tools. Further research, data analysis, and empirical testing will refine their value before effective implementation can occur.

Despite advantages like enhanced sensitivity, specificity, and diagnostic efficiency, machine learning has limitations worth noting. These include reliance on large datasets, low interpretability, dependence on specific algorithms and technologies, privacy concerns, feature extraction uncertainties, and other issues [43,45]. Future research should address opportunities for integrating multiple types of data sources, improving model interpretability, developing intelligent diagnostic models, creating large-scale specialized databases for tuberculosis, and establishing standardized clinical schemas for diagnosis and treatment of multidrug-resistant tuberculosis.

Bioinformatic analyses revealed that most differentially expressed genes were associated with immune responses, inflammation processes, and cell cycle regulation. Subsequent verification using quantitative PCR confirmed four differentially expressed genes—NEMF, ASUN, DHX29, and PTPRC—as potential biomarkers for identifying active and latent tuberculosis infections. Receiver operating characteristic (ROC) curve analysis indicated that PTPRC expression levels distinguish patients with active tuberculosis from healthy individuals, while ASUN distinguishes latent tuberculosis from health status. Conversely, DHX29 can identify latently infected people among those with active disease or healthy controls. To evaluate the potential application of these biomarkers as auxiliary diagnostic tools, we created classification models based on these candidate biomarkers and found that a naive Bayes classifier built upon ASUN, DHX29, and PTPRC provides optimal performance.

Our findings suggest that blood gene expression profiles can not only detect active forms of tuberculosis but also differentiate patients with latent infections from healthy individuals. Validation of our computational model in larger cohorts would confirm reliability of these biomarkers and facilitate development of affordable and sensitive platforms for molecular tuberculosis diagnostic [46].

People infected with *Mycobacterium tuberculosis* (MTB) may either eliminate the pathogen, develop latent infection, or progress to active disease. However, factors influencing pathogen elimination, transition to latency, and progression to active disease remain poorly understood. Herein, we attempted to employ a genome-wide transcriptional profiling approach to identify immune factors related to MTB infection and novel biomarkers capable of distinguishing active disease from latent infection.

Using microarrays, we comprehensively analyzed transcriptional differences in peripheral blood mononuclear cells (PBMCs) stimulated with purified protein derivative (PPD) in twelve participants divided into three groups: tuberculosis patients (TB), individuals with latent tuberculosis infection (LTI), and healthy control group members (HCG) (four per group). Transcriptome profiling of 506 differentially expressed genes correctly classified participants into three clusters. Additionally, two distinct signatures consisting of 55 and 229 transcripts were identified for tuberculous infection (TB + LTI) and active disease (TB), respectively. Quantitative real-time polymerase chain reaction (RT-qPCR)-based validation involving 83 subjects confirmed patterns of differential expression for 81% of the genes initially detected through microarray analysis. Decision tree analysis demonstrated that three genes—CXCL10, ATP10A, and TLR6—could help distinguish TB patients from those with latent tuberculosis infection. An additional evaluation of diagnostic capacity was conducted in 36 participants, yielding sensitivity of 71% and specificity of 89%. Transcriptional profiling of PBMCs induced by paraffin extract uncovered distinctive gene expression patterns corresponding to differing infectious statuses, offering new insights into human immune responses to MTB. Furthermore, this study suggests that the combination of CXCL10, ATP10A, and TLR6 might serve as novel biomarkers for differentiating tuberculosis from latent tuberculosis infection [47].

PPD contains a heterogeneous mixture of mycobacterial antigens shared by *Mycobacterium tuberculosis*, Bacillus Calmette–Guérin (BCG), and multiple environmental non-tuberculous mycobacteria. As a result, PPD-based assays lack specificity and cannot reliably discriminate between prior BCG vaccination, exposure to environmental mycobacteria, and true M. tuberculosis infection. This limitation provided the rationale for the development of interferon-gamma release assays (IGRAs) based on RD1-encoded antigens, such as ESAT-6 and CFP-10, which are absent from BCG strains and most non-tuberculous mycobacteria.

*Mycobacterium tuberculosis* can cause either active disease or latent infection in humans. However, factors promoting maintenance of latent infection and disease progression remain inadequately characterized. We employed RNA sequencing (RNA-seq) to uncover factors linked to *M. tuberculosis* state and a novel gene signature enabling differentiation between active disease and latent infection. Through RNA-seq, we characterized transcriptional differences in PBMCs stimulated with purified protein derivative (PPD) across three groups: active tuberculosis patients (AT), persons with latent tuberculosis infection (LTI), and uninfected control group members (CG). Collectively, 401 differentially expressed genes permitted clustering of participants into three categories. Validation by quantitative reverse-transcription PCR (RT-qPCR) confirmed significant expression differences in TNFRSF10C, IFNG, PGM5, EBF3, and A2ML1 between AT and LTBI groups. Additional clinical validation assessed diagnostic efficacy of these five biomarkers across 130 participants. A triplet set comprising TNFRSF10C, EBF3, and A2ML1 accurately classified 91.5% of patients with high sensitivity (86.2%) and specificity (94.9%). Diagnosing efficacy of the triplet gene set was further validated in a clinical sample of 147 suspected AT cases, resulting in sensitivity and specificity rates of 82.4% and 92.4%, respectively. Overall, we observed unique gene expression patterns in PBMCs depending on M. tuberculosis infection status. Moreover, we established a triplet gene set capable of distinguishing active tuberculosis from latent tuberculosis infection, which holds promise for facilitating rapid diagnosis and timely intervention towards better disease management [48].

Collectively, these studies illustrate the broad methodological diversity underlying biomarker signature construction. Multi-parametric ML-derived biosignatures consistently outperform single-analyte markers and often exceed the diagnostic performance of standard IGRAs in distinguishing LTBI from ATB [36]. Notably, the most reproducible signals across platforms include interferon-inducible chemokines (CXCL10/IP-10, occasionally CXCL9), innate immune mediators, vascular activation markers (e.g., VEGF), and enzymatic activities reflecting cellular immunity (e.g., ADA2). These soluble biomarkers align closely with transcriptomic signatures—particularly those enriched for IFN-related modules—enhancing their biological plausibility and cross-cohort reproducibility.

While ML methods offer enhanced sensitivity, specificity, and efficiency, they present limitations: requirement for large datasets, reduced interpretability, dependence on computational platforms, data privacy concerns, and uncertainty in feature selection. Future research should prioritise integration of multimodal datasets, improved model explainability, the development of intelligent diagnostic systems, creation of large-scale TB biobanks, and standardisation of clinical diagnostic algorithms. ML-driven early detection of TB and LTBI represents a promising path toward reducing LTBI progression, thereby contributing to WHO targets for TB elimination by 2035.

## 8. Biomarkers

To identify novel and clinically actionable blood-derived biomarkers, the authors optimised an integrative bioinformatic pipeline combining differentially expressed gene (DEG) analysis, gene co-expression network mining, and short time-series expression analysis to interrogate published whole-blood transcriptomic datasets from patients with tuberculosis available in the GEO repository. The diagnostic performance of candidate biomarkers was subsequently assessed in independent datasets and in blood samples from Chinese patients using quantitative real-time PCR (qRT-PCR). This approach identified four genes—UBE2L6 (ubiquitin/ISG15-conjugating enzyme E2 L6), BATF2 (basic leucine zipper ATF-like transcription factor 2), SERPING1 (C1 inhibitor), and VAMP5 (vesicle-associated membrane protein 5)—with high diagnostic value for active tuberculosis.

On average, transcriptional levels of these four genes achieved 88% sensitivity and 78% specificity in diagnosing active TB. The highest sensitivity (up to 100%) was obtained when BATF2 and VAMP5 were used in combination, whereas the greatest specificity (89.5%) was achieved using SERPING1, UBE2L6, and VAMP5. These values markedly exceeded the performance of the T-SPOT.TB assay in the same patient cohort (75.3% sensitivity and 69.1% specificity).

Interestingly, this four-gene signature also demonstrated utility in monitoring treatment response and discriminating active TB from LTBI. The available evidence therefore suggests that these biomarkers may be more efficient and clinically preferable than current IGRAs for tuberculosis diagnostics [12,14].

To identify clinically deployable blood-based biomarkers, the study by Gong et al. proposed an integrative analytic pipeline for whole-blood transcriptomes (GEO), combining three complementary strategies:

(i) identification of differentially expressed genes;
(ii) co-expression network analysis to capture stable modules associated with infection status; and
(iii) short time-series analysis to track dynamic transcriptomic trajectories during treatment.

This design enables simultaneous capture of both static inter-group differences and temporal variation, which is essential for distinguishing LTBI from ATB and for treatment monitoring. Candidate biomarkers were then transferred to a targeted qRT-PCR platform and validated in independent clinical samples.

Integration of these datasets highlighted a compact four-gene panel—UBE2L6, BATF2, SERPING1, VAMP5—with strong diagnostic performance. Validation in independent datasets and in a clinical cohort of Chinese patients demonstrated:

- Mean sensitivity ~88% and specificity ~78% for ATB;
- BATF2 + VAMP5 achieving 100% sensitivity;
- SERPING1 + UBE2L6 + VAMP5 achieving 89.5% specificity.

These metrics substantially exceeded those of T-SPOT.TB within the same cohort, underscoring the diagnostic value of host-driven transcriptomic markers.

A key practical strength of this panel is its dual utility: in addition to discriminating ATB from LTBI, the markers exhibited dynamic changes during anti-TB therapy, making them suitable for treatment monitoring. Biologically, the panel aligns with established TB-related immune pathways:

- UBE2L6 and BATF2 reflect interferon-driven innate immune programmes;
- SERPING1 regulates complement activation and inflammation;
- VAMP5 participates in vesicular trafficking, antigen presentation, and mediator secretion.

A clinically translatable pipeline for such signatures includes:

1. Standardised pre-analytics (matrix handling, RNA stabilisation);
2. QRT-PCR with primer-efficiency control and ΔCt normalisation;
3. Robust feature selection (regularisation, ensemble stability analysis);
4. Interpretable modelling (regularised logistic regression or decision tree) with probability calibration;
5. Validation in independent cohorts.

Recommended reporting includes AUC, sensitivity/specificity, calibration, and—for longitudinal data—the evolution of signature scores and time-dependent AUC.

Interestingly enough, this gene set can assess the effectiveness of anti-tuberculosis therapy and distinguish active tuberculosis from latent tuberculosis infection. Data showed that these four biomarkers could be more effective and preferable than IGRAs in diagnosing tuberculosis [49].

To search for clinically applicable blood-derived biomarkers, ref. [50] proposed an integrative pipeline analyzing published whole-blood transcriptomes (GEO), combining three complementary approaches: (i) identification of differentially expressed genes, (ii) coexpression network analysis to select stable modules associated with infection status, and (iii) short-term trajectory analysis to track dynamic changes during treatment. This design allows capturing both “snapshot” differences between groups and their temporal trajectories, essential for differentiating LTI/ATB and monitoring therapy. Candidate markers then undergo translation onto a target platform and clinical validation by quantitative real-time PCR (qRT-PCR) on independent patient samples.

The integrated data highlighted a compact quartet of genes—UBE2L6 (ubiquitin/ISG15-conjugating enzyme E2 L6), BATF2 (AP-1-like family transcription factor), SERPING1 (complement component inhibitor), and VAMP5 (vesicular membrane protein 5)—possessing high diagnostic value in active tuberculosis. Independent validations and clinical cohort studies in Chinese patients exhibited average sensitivity (88%) and specificity (78%) for ATB diagnosis based on transcription of these genes; notably, the BATF2 + VAMP5 duo ensured up to 100% sensitivity, whereas the SERPING1 + UBE2L6 + VAMP5 trio reached up to 89.5% specificity. Performance significantly exceeded that of T-SPOT.TB in the same cohort (sensitivity 75.3%, specificity 69.1%), emphasizing the informational value of host-driven transcriptomic signals for fast diagnostics [51,52].

An important practical feature is dual utility: beyond diagnosing ATB, these markers show variability over anti-tuberculous therapy and ability to distinguish ATB from LTI, making them candidates for tasks of differential diagnosis of infection status and therapeutic monitoring. Biologically, the panel converges with known immune pathways in TB: UBE2L6 and BATF2 reflect IFN-dependent programs of innate immunity; SERPING1 relates to complement regulation and inflammation; VAMP5 participates in vesicle trafficking crucial for antigen presentation and mediator secretion.

For clinical translation, the following workflow is exemplary: standard preanalytics (matrix stabilization), qRT-PCR with primer-efficiency control and normalization (ΔCt), selection of minimized subsets (regularization/bootstrap-stabilized ensembles), construction of interpretable models (regularized logistic regression/decision trees) calibrated for probabilistic outputs, and validation on independent cohorts. It is advisable to report AUC, sensitivity/specificity, calibration metrics, and, for longitudinal data, dynamics of the signature and time-dependent AUC [53].

Limitations of current evidence base include regional homogeneity of main clinical samples (China), potential discrepancies in preanalytical steps and platforms, and risk of metric optimism due to small subgroup sizes. External multicentric validations (different geographies, HIV coinfection, comorbidities), harmonization of SOPs and cut-offs, economic evaluations, and tests in actual diagnostic workflows are needed to increase generalizability. Nonetheless, the presented pipeline—from wide integrative searches in GEO to a concise qRT-PCR signature with clinical validation—demonstrates a practical pathway for generating quick and accurate tests potentially superior to IGRAs, accelerating diagnosis and supporting decisions about initiating therapy in regions with high prevalence. Proteomics, despite being directly relevant to disease phenotype, faces technological and analytical constraints limiting its use with ML. Key challenges include the vast concentration range of blood proteins (spanning 10–12 orders of magnitude), complicating simultaneous detection of highly abundant and rare markers (like cytokines); preanalytic influences such as storage conditions, posttranslational modifications, and proteolytic activity also affect outcomes.

ML approaches in proteomics yield promising results. Ensemble methods (Boosting, Random Forests) applied to large multiplexed panels of cytokines and chemokines (up to 100 markers) exhibit strong capability to differentiate ATB/LTI with AUC > 0.90 at the expense of reduced interpretability. Addressing this limitation and enhancing model robustness increasingly involves multimodal integration [49].

Multimodal integration represents the future of precise LTI diagnostics. Combining transcriptomic signatures reflecting genetic programming with proteomic biomarkers representing functional activity enables ML models to construct a more comprehensive representation of pathogenesis. Potential gains lie in increased predictive strength and resilience to variations since the model does not rely solely on one type of data prone to external noise. Current developments focus on designing integrative pipelines capable of identifying synergistic biomarker modules.

Limitations of the current evidence base include demographic homogeneity (primarily China), variation in pre-analytical procedures and platforms, and the risk of optimistic performance estimates in small subgroups. Enhancing generalisability will require multicentre validation across diverse geographic regions, inclusion of cohorts with HIV co-infection and major comorbidities, harmonisation of SOPs and thresholds, and economic evaluation within real-world diagnostic workflows. Nonetheless, this end-to-end strategy—from broad integrative discovery to compact qRT-PCR signatures with clinical validation—demonstrates a practical trajectory for generating rapid, accurate diagnostics that can complement or outperform IGRAs, expedite clinical decision-making, and support TB control efforts in high-burden regions.

### 8.1. Transcriptomic Biomarker Studies (Summaries of Cited Works)

*Study 1: NEMF, ASUN, DHX29, and PTPRC as diagnostic candidates.*

Bioinformatic analysis identified several differentially expressed genes associated with immune responses, inflammation, and cell-cycle regulation. Subsequent RT-PCR validation confirmed four genes—NEMF, ASUN, DHX29, and PTPRC—as potential biomarkers discriminating active and latent TB. ROC analysis demonstrated that PTPRC distinguished ATB from healthy controls, ASUN distinguished LTBI from healthy controls, and DHX29 discriminated LTBI from both ATB and uninfected individuals. A naïve Bayes classifier using ASUN, DHX29, and PTPRC demonstrated optimal performance. Larger-cohort validation is expected to support development of cost-effective and sensitive molecular diagnostics [42].

*Study 2: CXCL10, ATP10A, and TLR6 signature.*

Whole-genome microarray profiling of PPD-stimulated PBMCs from 12 subjects (ATB, LTBI, and healthy controls) identified 506 differentially expressed genes, enabling clear three-group clustering. Gene signatures comprising 55 and 229 transcripts were associated with TB infection and active disease, respectively. Validation via qPCR in 83 participants confirmed 81% of microarray-identified genes. Decision-tree analysis revealed CXCL10, ATP10A, and TLR6 as discriminatory biomarkers for ATB vs. LTBI, achieving 71% sensitivity and 89% specificity in validation [43].

*Study 3: TNFRSF10C, EBF3, A2ML1 signature via RNA-seq.*

RNA-seq profiling of PPD-stimulated PBMCs identified 401 differentially expressed genes separating ATB, LTBI, and uninfected individuals into distinct clusters. Validation confirmed differential expression of TNFRSF10C, IFNG, PGM5, EBF3, and A2ML1. A three-gene combination (TNFRSF10C, EBF3, A2ML1) correctly classified 91.5% of participants (sensitivity 86.2%; specificity 94.9%) and demonstrated strong performance in an independent clinical cohort (sensitivity 82.4%; specificity 92.4%) [44].

### 8.2. Proteomics and Machine Learning

Although proteomics directly reflects disease phenotype, it faces several key technological and analytical challenges relevant to ML-based applications. One major limitation is the extremely broad dynamic range of protein concentrations in blood (up to 10–12 orders of magnitude), which complicates simultaneous detection of highly abundant proteins and low-abundance biomarkers such as cytokines. Pre-analytical factors—including sample handling, storage, post-translational modifications, and proteolytic activity—further influence data integrity.

ML approaches in proteomics have shown promising results. Ensemble methods such as boosting and random forests, when applied to multiplex cytokine and chemokine panels containing up to 100 proteins, have demonstrated strong discriminatory power for ATB versus LTBI (AUC > 0.90). However, this performance is often achieved at the expense of interpretability. To address this limitation and enhance model robustness, multi-omics integration is gaining increasing importance.

### 8.3. Multi-Omics Integration (Multimodal ML)

Multimodal ML represents the future of precision diagnostics for LTBI. Combining transcriptomic signatures (reflecting regulatory gene programmes) with proteomic biomarkers (reflecting functional immune activity) enables ML models to generate a more comprehensive representation of disease biology. This approach offers improved predictive power and greater robustness, since the model does not rely on a single data modality that may be sensitive to external noise.

Rapid development is currently underway to design integrative pipelines capable of identifying synergistic biomarker modules across omics layers. Such multi-dimensional signatures have the potential to substantially improve the accuracy, reproducibility, and clinical translatability of LTBI diagnostics.

## 9. Cellular Biomarkers for Differential Diagnosis of ATB and LTBI

Circulating blood lymphocytes play a critical role in host defence against MTB through a broad range of effector and regulatory functions, including cytokine and chemokine production and the generation of MTB-specific antibodies [54,55]. Therefore, analyzing alterations in T- and B-cell subpopulation profiles across different disease states represents a highly promising strategy for identifying novel biomarkers capable of distinguishing active tuberculosis (ATB) from latent tuberculosis infection (LTBI).

Analysis of peripheral blood samples from individuals with ATB and LTBI demonstrated that the monocyte-to-lymphocyte ratio (MLR) (AUC = 0.856; cut-off > 0.293; sensitivity 92.31% [74.87–99.05], specificity 74.07% [53.71–88.89]) and the neutrophil-to-lymphocyte ratio (NLR) (AUC = 0.839; cut-off > 2.867; sensitivity 76.92% [56.35–91.03], specificity 88.89% [70.84–97.65]) were significantly elevated during active disease compared with LTBI [56]. Comparable discriminative performance was observed for Th1–Th17 or Th17.1 cells with a CD4^+^CD196^+^CD183^+^ phenotype (AUC = 0.808; cut-off < 3.210; sensitivity 90.00% [68.30–98.77], specificity 68.42% [43.45–87.42]). Notably, Th17 cells and their subsets have well-established roles in TB immunopathogenesis [56]. CD161, another key marker of IL-17-producing cells [57], also demonstrated diagnostic potential. Zhang, Q. showed that CD3^+^CD161^+^, CD3^+^CD4^+^CD161^+^, and particularly CD3^+^CD8^+^CD161^+^ T cells discriminate ATB from LTBI [55]. The proportion of CD161^+^ CD8^+^ T cells exhibited especially high diagnostic accuracy (AUC = 0.933 [0.877–0.989]; sensitivity 88.89% [70.84–97.65], specificity 94.12% [87.64–97.81]) [3].

Additional circulating lymphocyte subsets have also been proposed as potential biomarkers for ATB [47,58]. Compared with healthy controls, individuals with ATB showed reduced frequencies of Vδ2^+^ and Vδ2^−^ T cells, invariant natural killer T cells (iNKT), and CCR4^+^ memory regulatory T cells (Tregs), whereas Th2 cells and total Tregs were increased. Liu et al. further demonstrated that sputum culture-negative TB patients exhibited decreased frequencies of central memory (CD45RA^−^CD27^+^) Vδ2 T cells compared with LTBI individuals, suggesting an essential role of this subset in IFN-γ production and infection control [59]. The diagnostic performance of CD45RA^−^CD27^+^ Vδ2 T cells (AUC = 0.839; sensitivity 81.0%, specificity 81.2%) supports their utility in distinguishing ATB from LTBI.

In the study of peripheral blood samples obtained from patients with active tuberculosis (ATB) and latent tuberculosis infection (LTBI), it has been shown that monocyte-to-lymphocyte ratio (MLR) and neutrophil-to-lymphocyte ratio (NLR) are significantly elevated in cases of active TB compared to LTBI (Monocyte-to-Lymphocyte Ratio: AUC = 0.856, cutoff value > 0.293 with sensitivity and specificity of 92.31% (74.87–99.05) and 74.07% (53.71–88.89); Neutrophil-to-Lymphocyte Ratio: AUC = 0.839, cutoff value > 2.867 with sensitivity and specificity of 76.92% (56.35–91.03) and 88.89% (70.84–97.65)) [54]. Equally significant prognostic importance for differentiation between active TB and LTBI was found when determining levels of Th1-Th17 or Th17.1 cells with phenotype CD4^+^CD196^+^CD183^+^ (AUC = 0.808, cutoff value < 3.210 with sensitivity and specificity of 90.00% (68.30–98.77) and 68.42% (43.45–87.42)). It should be noted that Th17 cells and their individual subpopulations play a crucial role in the pathogenesis of tuberculosis infection [3].

Another important marker of IL-17-producing cells is molecule CD161 [58]. According to Yang et al.’s research, CD3^+^CD161^+^, CD3^+^CD4^+^CD161^+^, and CD3^+^CD8^+^CD161^+^ T cells can be applied for differential diagnostics of active TB and LTBI [3]. Thus, the level of CD161^+^ CD8^+^ T cells very efficiently separates these patient groups (AUC = 0.933 (0.877–0.989) with sensitivity and specificity of 88.89% (70.84–97.65) and 94.12% (87.64–97.81)), while lower values were observed for populations of CD3^+^CD161^+^ and CD3^+^CD4^+^CD161^+^ T cells. Furthermore, certain circulating lymphocyte subsets have also been identified among patients with ATB that could serve as potential diagnostic markers [60,61]. Compared to healthy controls, patients with ATB had decreased levels of Vδ2^+^ T cells, Vδ2^−^ T cells, invariant natural killer T cells (iNKT), and CCR4^+^ memory Tregs, whereas Th2 cells and Tregs increased in circulation. Additionally, according to Liu et al., central memory (CD45RA-CD27^+^) Vδ2 T cells showed reduced levels in the bloodstream of patients with sputum culture-negative tuberculosis compared to those with LTBI, indicating the significance of these cells in producing IFN-γ and controlling infection progression [62]. Moreover, the area under the curve (AUC) for CD45RA-CD27^+^Vδ2 T cells was 0.839 with sensitivities of 81.0% and specificities of 81.2%. Another important subset of T cells involved in effective protection against *MTB* are mucosal-associated invariant T (MAIT) cells, which perform a wide spectrum of effector functions related to production of effector cytokines and activation of various cells at the site of pathogen invasion [63].

Circulating levels of CD8^+^CD161^+^TCR-Vα7.2^+^ MAIT cells in LTBI were significantly higher than those seen in patients with active TB, making them promising candidates for differential diagnostics (AUC = 0.716, cutoff value < 4.535 with sensitivity and specificity of 82.35% (56.57–96.20) and 64.71% (38.33–85.79)) [54]. Furthermore, MAIT cells (CD3^+^CD4^−^Vα7.2^+^CD161high T cells) were found to be fewer in number in peripheral blood samples taken from children with active TB versus children with LTBI and healthy controls [64]. Therefore, determination of MAIT cells might serve as a universal marker across different age groups. Beyond identification of leukocyte subsets in whole blood samples from patients with active TB and LTBI, several approaches aimed at activating MTB-specific T cells in vitro are widely employed for differential diagnostics. For instance, CD69 serves as one of the early activation markers whose membrane expression increases shortly after recognition of a specific antigen by CD4^+^ and CD8^+^ T-lymphocyte receptors [65].

Previously, it was established that the level of CD69^+^ CD4^+^ T cells rose in patients with active TB and individuals with clinically inactive TB relative to BCG-vaccinated healthy individuals who tested negatively for tuberculin skin tests [66]. More recently, Yang et al. demonstrated that assessment of CD69^+^ CD4^+^ T-cell levels not only allows separation of ATB and LTBI (AUC value for CD69 on T cells was 0.74), but also enables differentiation between ATB and healthy controls (HC) or non-TB infections with excellent diagnostic utility (AUC = 0.91 and AUC = 0.87, respectively) [67]. Furthermore, in children with active TB, the frequency of CD69^+^ MAIT cells was higher than in children with LTBI (20.7 vs. 7.5%, *p* < 0.001) and healthy control children (9.1%, *p* = 0.007) [64], thus enabling its use in pediatric assessments. Although there are data showing no statistically significant differences in CD69 expression by CD4+ and CD8^+^ T-lymphocytes between patients with active TB and latent infection [68]. It is worth mentioning studies focused on in vitro stimulation of antigen-specific T cells from MTB-infected patients using mixtures of peptides such as ESAT-6/CFP-10, where substantial differences in cytokine production and cellular activation markers were detected between patients with ATB and LTBI. Specifically, levels of MTB-specific IFN-γ^+^CD4^+^ T cells expressing activation markers like CD38 or HLA-DR or proliferation marker Ki-67 were higher in patients with ATB compared to LTBI (cutoff values of 18% for CD38^+^IFN-γ^+^, 60% for HLA-DR^+^IFN-γ^+^, and 5% for Ki-67^+^IFN-γ^+^ CD4^+^ T cells, achieving 100% specificity and over 96% sensitivity) [69].

Similarly high relevance for separating ATB and LTBI was found when measuring levels of IL-2^+^IFNγ^+^ T cells following in vitro stimulation of peripheral blood samples with BCG and purified protein derivative (PPD) [70]. Later studies revealed intriguing findings regarding evaluation of HLA-DR expression on BCG-specific IFNγ^+^TNF^+^IL2– CD4^+^ T-cells, yielding 95% specificity and 95% sensitivity when distinguishing ATB and LTBI patients [71]. It must be acknowledged that setting up this type of reaction in vitro is complex, time-consuming, requires highly skilled personnel, and sophisticated equipment to achieve accurate results, thereby limiting widespread application in clinical practice. One more approach for detecting MTB-specific T cells in response to in vitro stimulation involves assessing co-expression of multiple specific markers appearing on the surface of activated T cells (activation-induced markers, AIM) [72]. For example, quantification of CD25^+^CD134^+^ CD8^+^ T cells upon activation with ESAT-6 or CFP-10 enabled discrimination between active TB and uninfected patients with a sensitivity and specificity of 68% (95% CI 46.5–85%) and 100% (95% CI 82.3–100%), respectively [73].

In the case of CD25^+^CD134^+^ CD4^+^ T cells, sensitivity reached 100% (95% CI 86.2–100%) and specificity achieved 100% (95% CI 82.3–100%) for diagnosing active TB. Subsequently, it was shown that stimulating T cells in vitro with ESAT-6 and CFP-10 followed by evaluating CD25, CD69, and CD134 expression on CD4^+^ T cells proved effective for distinguishing TB-infected patients from healthy controls [74]. In terms of CD25^+^CD134^+^ CD4^+^ T cells, AUC = 0.93 corresponded to sensitivity and specificity rates of 86.7% (72.5–94.5%) and 83.3% (68.0–92.5%), respectively, while for CD69^+^CD134^+^ CD4^+^ T-cell population, AUC = 0.91 yielded similar sensitivity and specificity metrics. However, measuring CD25^+^CD134^+^ and CD69^+^CD134^+^ CD4^+^ T-cell levels did not prove suitable for differentiating between active and latent TB patients. It is essential to mention the method developed by Luo et al. for distinguishing patients with ATB from LTBI individuals based on concurrent evaluation of cell-surface activation antigens (HLA-DR) and intracellular cytokine synthesis (TNF-α and IL-2) [50]. Results indicated that presence of HLA-DR on TNF-α^+^IL-2^+^ CD4^+^ T-cells generated an AUC of 0.901 (95% CI, 0.833–0.969) with a sensitivity of 93.75% (95% CI, 79.85–98.27%) and specificity of 72.97% (95% CI, 57.02–84.60%) when employing a threshold of 44% HLA-DR^+^ cells. Further investigation involving larger cohorts confirmed that HLA-DR expression by MTB-specific IFN-γ^+^TNF-α^+^ CD4^+^ T-cells separated ATB patients from LTBI individuals with an AUC of 0.917 (95% CI, 0.856–0.977), demonstrating sensitivity and specificity of 91.89% (95% CI, 78.70–97.21%) and 63.89% (95% CI, 47.58–77.53%) [75]. Lastly, a recent innovation introduced the blood-based TB-Flow Assay, capable of differentiating pulmonary and extrapulmonary TB diseases from TB infection within 24 h post-stimulation with PPD and ESAT-6/CFP-10 [75]. Upon in vitro stimulation with PPD and ESAT-6/CFP-10, frequencies of CD38^+^CD154^+^, HLA-DR^+^CD154^+^, and Ki-67^+^CD154^+^ cells within CD154^+^CD4^+^ T-cells and total CD4^+^ T-cells were assessed. Then, twelve flow cytometric parameters exceeding respective cutoff points received a score of 1, otherwise receiving a score of 0. Summing these scores yields the ‘TB-Flow Score’, ranging from 0 to 12. Optimal ‘TB-Flow Score’ cutoff point for distinguishing TB infection and TB disease in patients was set at 3.5 via ROC curve analysis (AUC 0.978, 95% CI 0.945–1.000). Ultimately, the diagnostic sensitivity and specificity of the TB-Flow Assay were reported as 93.6% (95% CI 82.5–98.7%) and 97.1% (95% CI 84.7–99.9%), respectively.

Mucosal-associated invariant T (MAIT) cells, which exert broad effector functions including cytokine secretion and activation of local immune responses [49], represent another important population involved in MTB control. Circulating CD8^+^CD161^+^TCR-Vα7.2^+^ MAIT cell frequencies were significantly higher in LTBI than ATB, providing moderate discriminative capacity (AUC = 0.716; cut-off < 4.535; sensitivity 82.35% [56.57–96.20], specificity 64.71% [38.33–85.79]) [45]. Similar trends were observed in paediatric cohorts, where children with ATB exhibited reduced frequencies of CD3^+^CD4^−^Vα7.2^+^CD161^high MAIT cells compared with LTBI and healthy controls [62]. These findings support the potential of MAIT-cell monitoring as a biomarker applicable across age groups.

Beyond quantification of lymphocyte subsets in peripheral blood, in vitro activation assays targeting MTB-specific T cells are widely employed for differential diagnosis. CD69 is a well-established early activation marker upregulated on CD4^+^ and CD8^+^ T cells shortly after antigen recognition [49]. Early studies demonstrated that CD69^+^CD4^+^ T cells were more frequent in individuals with active or inactive TB than in BCG-vaccinated tuberculin-negative controls [48]. Recent work by Yang et al. showed that CD69 expression on CD4^+^ T cells discriminates ATB from LTBI (AUC = 0.74) and from both healthy controls and non-TB infections (AUC = 0.91 and 0.87, respectively) [57]. Nonetheless, some studies reported no significant differences in CD69 expression on CD4^+^ or CD8^+^ T cells between ATB and LTBI [53], indicating variability across cohorts.

A combined phenotypic–functional strategy was explored by Luo et al., who assessed co-expression of HLA-DR on TNF-α^+^IL-2^+^ CD4^+^ T cells. This yielded an AUC of 0.901 (sensitivity 93.75%, specificity 72.97%) using a 44% positivity threshold. In an expanded cohort, HLA-DR expression on MTB-specific IFN-γ^+^TNF-α^+^ CD4^+^ T cells provided an AUC of 0.917 with sensitivity 91.89% and specificity 63.89% [26].

Most recently, a blood-based TB-Flow Assay was developed to differentiate pulmonary and extrapulmonary TB disease from TB infection within 24 h of stimulation with PPD or ESAT-6/CFP-10 [3]. Frequencies of CD38^+^CD154^+^, HLA-DR^+^CD154^+^ and Ki-67^+^CD154^+^ CD4^+^ T cells were quantified, and 12 diagnostic parameters were assigned binary values to generate a composite “TB-Flow Score” (0–12). A threshold of 3.5 optimally distinguished TB disease from infection (AUC = 0.978 [95% CI 0.945–1.000]). The assay demonstrated high diagnostic sensitivity (93.6% [82.5–98.7]) and specificity (97.1% [84.7–99.9]) [76].

## 10. Neural Network Technologies for LTBI Diagnostics

Neural network technologies have become an indispensable tool for addressing complex diagnostic tasks, gradually replacing classical machine-learning approaches in domains characterized by high-dimensional data and intricate spatiotemporal patterns. Their primary advantage is the ability to automatically extract relevant features and model complex nonlinear interactions, which is particularly critical for the challenging task of diagnosing latent tuberculosis infection (LTBI). Recent systematic reviews highlight a steady increase in publications in which neural networks are used either as standalone classifiers or as tools for generating informative feature embeddings to be further analyzed using traditional algorithms [14].

Among the most successful applications of neural network technologies in tuberculosis diagnostics is computer vision for immunological image analysis. Luo et al. proposed a two-stage convolutional neural network (CNN) architecture for processing T-SPOT.TB assay images [77]. The CNN is trained directly on spot-based assay images to automatically extract quantitative and spatial characteristics, which are subsequently used in a logistic regression model. This approach improves diagnostic resolution for differentiating active tuberculosis (ATB) from LTBI compared with standard manual evaluation.

Modern studies analyzing chest X-ray (CXR) imaging have adopted hybrid deep-learning architectures. A recent study based on combined EfficientNet and MLP-Mixer models demonstrated competitive performance, achieving an accuracy of up to 96.3%, sensitivity of 95.9%, and specificity of 96.6% in distinguishing active from non-active disease [78]. In clinical practice, such neural-network-based systems are essential for early screening, enabling efficient triage of patients with clear radiographic signs of active disease or visual markers associated with non-active but high-risk tuberculosis.

In the field of omics analysis, neural networks are primarily used for dimensionality reduction and identification of latent biological representations [79,80]. Autoencoder-based architectures are frequently employed for compressing transcriptomic data and generating feature embeddings, which efficiently capture the underlying biological structure and enable identification of high-risk LTBI molecular subtypes.

Another promising direction is the development of multimodal ensembles capable of integrating information from multiple heterogeneous sources. Modern neural-network architectures—particularly Transformer models incorporating attention mechanisms—enable effective fusion of transcriptomic profiles, immunological assay results, and radiological features. Such multimodal approaches may substantially enhance diagnostic accuracy and support risk stratification in both clinical and population-level LTBI screening (Table 4).

This table summarises deep-learning and neural-network models used for LTBI-associated diagnostic tasks, including interpretation of T-SPOT.TB results, radiological assessment using chest X-ray imaging, transcriptomic dimensionality reduction, and multimodal integration of heterogeneous datasets. For each model, the underlying architecture, data source, performance metrics, and potential clinical applications are described.

## 11. Limitations, and Future Directions

Advances in omics technologies combined with state-of-the-art machine-learning (ML) methods are opening a new era in LTBI diagnostics, shifting the focus from non-specific clinical and radiological criteria towards precise molecular signatures. Transcriptomic and proteomic signatures derived from host immune responses show substantial promise for distinguishing ATB from LTBI, with reported AUC values ranging from 0.85 to 0.98. Interferon-dependent genes (e.g., GBP2, CXCL10, IFITM3) are consistently implicated, emphasising the central role of type I/II IFN-mediated responses in ATB pathogenesis. Successful translational pathways—from broad omics screens to concise, clinically validated qRT-PCR signatures—illustrate a realistic trajectory towards clinical application [47,81]. Despite significant progress, several key limitations of the current evidence base and ML approaches need to be addressed to ensure robustness and generalisability of proposed diagnostic tools.

### 11.1. Reproducibility and Validation Challenges

Many published models have been evaluated only in internal or limited external cohorts. The absence of multi-centre, multinational validation reduces confidence in their transportability to diverse populations, including individuals with HIV co-infection or those living in regions with high prevalence of non-tuberculous mycobacteria. Ensuring reliability requires adherence to TRIPOD and PROBAST standards, along with public release of “frozen” algorithm versions for independent replication [82,83].

### 11.2. Data Heterogeneity and Batch Effects

Variation in sample-processing methods (whole blood vs. PBMC), assay platforms (microarray vs. RNA-seq; ELISA vs. Luminex), and stimulation protocols (PPD vs. ESAT-6/CFP-10) introduce substantial batch effects that ML models may incorrectly learn as disease-relevant signals. Proteomics faces additional challenges due to the extreme dynamic range of protein concentrations in blood. Addressing these issues requires stringent harmonisation of standard operating procedures (SOPs) and application of integrative batch-correction approaches to improve cross-cohort comparability [3,59].

### 11.3. Accuracy–Interpretability Trade-Off

Although ensemble ML approaches (e.g., boosting, random forests) often yield superior accuracy, their “black-box” nature limits clinical interpretability and may reduce clinicians’ trust. The application of explainable artificial intelligence (XAI) methods—including SHAP and LIME—has become an essential step for elucidating the contribution of individual biomarkers and supporting the rationale behind model predictions [84,85,86].

### 11.4. Predominant Focus on Diagnosis Rather than Prognosis

Most studies to date have concentrated on cross-sectional differentiation between ATB and LTBI. However, effective LTBI prevention requires longitudinal cohorts and models capable of predicting progression to active disease. Time-dependent performance metrics (e.g., time-dependent AUC) are essential for evaluating predictive utility, yet remain insufficiently explored [87,88,89]. Establishing long-term cohorts of individuals at elevated risk is critical for developing true prognostic tools (Table 5).

The principal methodological and translational limitations of existing LTBI diagnostic studies, including restricted external validation, batch effects, low interpretability of complex ML models, and the predominance of cross-sectional designs [87,90]. For each limitation, typical manifestations and their implications are summarised, together with recommended methodological solutions such as TRIPOD-compliant reporting, harmonisation of laboratory procedures, and incorporation of explainable AI techniques [91,92,93].

Future development of LTBI diagnostic technologies should prioritise the following areas:

#### 11.4.1. Multi-Omics Integration

Integration of complementary data modalities—transcriptomics, soluble immune mediators, cellular responses including single-cell RNA-seq—is expected to improve accuracy and robustness. ML models capable of extracting synergistic information across these layers are likely to be more resilient to biological and pre-analytical variability.

#### 11.4.2. Clinical Translation into Point-of-Care Formats

Successful diagnostic innovation requires efficient clinical translation [94,95] Key directions include:

- adapting high-performance molecular signatures into cost-effective and accessible platforms (e.g., qRT-PCR, multiplexed point-of-care assays);
- integrating diagnostic tools into clinical workflows using concise biomarker panels (3–10 markers), calibrated thresholds, and user-friendly scoring systems.

Beyond analytical performance, robust assessment of clinical usefulness is essential. Economic evaluation (cost–benefit analysis) and decision-curve analysis are required to quantify how ML-enabled diagnostics influence misclassification rates, patient management strategies, and downstream clinical outcomes [96].

## 12. Expanded Explanation of AI-Driven Analytical Frameworks

Recent advances in artificial intelligence have enabled the development of analytical frameworks capable of integrating high-dimensional transcriptomic data across multiple biological layers. These frameworks typically combine feature selection algorithms (e.g., LASSO, recursive feature elimination), dimensionality reduction techniques (e.g., principal component analysis, autoencoders), and supervised learning models such as random forests, support vector machines, and Bayesian classifiers. Such approaches facilitate the identification of compact gene signatures while minimising overfitting and enhancing interpretability.

Diagnostic model development using microarray or RNA-seq data generally follows a multistep pipeline that includes data normalisation, batch-effect correction, differential expression analysis, feature selection, and supervised model training. RNA-seq offers higher dynamic range and sensitivity compared with microarrays, whereas microarrays remain more cost-effective and widely available. Machine learning models trained on these platforms aim to identify transcriptomic signatures capable of distinguishing LTBI from ATB and, in some cases, predicting progression risk.

## 13. Confounding Effects of Other Pulmonary Infections and Inflammatory Conditions

A critical limitation of many machine learning–based biomarker studies for tuberculosis lies in the insufficient consideration of clinically relevant confounding conditions. Most published models focus primarily on binary discrimination between latent tuberculosis infection (LTBI) and active tuberculosis (ATB), while excluding patients with other pulmonary infections or non-tuberculous inflammatory diseases. This omission may substantially inflate apparent diagnostic performance and reduce real-world applicability.

Several non-tuberculous pulmonary infections and inflammatory conditions are associated with host immune responses that overlap with those observed in ATB. For instance, blood transcriptional profiles in pulmonary sarcoidosis share dominant interferon-inducible and proinflammatory pathways with TB signatures, and machine learning classifiers struggle to completely separate these conditions based on overlapping transcriptomic patterns alone. In comparative analyses, sarcoidosis and TB blood profiles cluster closely and share interferon and immune signalling pathways, highlighting the need for specific differential features in diagnostic models [97]. 

Analogous overlap has been observed in studies comparing multiple pulmonary conditions, where TB and sarcoidosis exhibited similar interferon-driven signatures that were distinct from profiles seen in pneumonia and lung cancer patients. These findings imply that purely host-derived inflammatory signatures may not be specific to TB in the presence of other granulomatous or inflammatory lung diseases. 

Viral respiratory infections, including SARS-CoV-2 (COVID-19), also share transcriptional perturbations with TB. Meta-analyses demonstrate significant immunopathogenic overlap between COVID-19 and TB signatures, particularly in innate immune and interferon response pathways, suggesting that shared inflammatory responses may confound transcriptomic discrimination between these diseases [98,99].

Non-tuberculous mycobacterial (NTM) infections further complicate the picture. Emerging bioinformatic evidence indicates that transcriptomic markers can be associated with both NTM and TB, indicating potential confounding at the transcript level when models are trained without appropriate control groups [100]. 

In addition to infectious causes, chronic inflammatory lung conditions such as sarcoidosis, chronic obstructive pulmonary disease (COPD), interstitial lung diseases, and other forms of immune-mediated pneumonitis can exhibit systemic immune activation and altered cytokine profiles overlapping with TB-associated signatures, particularly those driven by generalised inflammatory processes. This potential overlap underscores the need for inclusion of diverse control phenotypes in ML training datasets to improve specificity

## 14. Discussion

In this review, we synthesised current evidence on transcriptomic, proteomic, and machine learning (ML)-driven biomarker research for differentiating latent tuberculosis infection (LTBI) from active tuberculosis (ATB), highlighting both advances and persistent challenges. Despite substantial progress in high-dimensional omics and computational methodologies, a number of critical gaps limit the translation of research findings into clinically actionable diagnostics.

Integrative analyses that combine transcriptomic and proteomic data with AI-based frameworks have demonstrated improved discrimination between active tuberculosis, LTBI and other conditions. For example, a recent multi-omics study identified plasma biosignatures, including lipid species such as PC (14:0_22:6), that distinguish active TB from LTBI and non-TB groups with AUCs up to ~0.90 in both discovery and validation cohorts, emphasising the value of metabolic and protein markers together with ML modelling strategies [turn0search0]. These approaches exemplify how multidimensional integration can leverage complementary biological signals, but they also expose significant methodological complexity.

Despite promising results, several limitations constrain the current evidence base. Most studies rely on relatively small discovery cohorts and geographically homogeneous populations, limiting generalisability. Proteomic biomarkers are affected by wide concentration ranges in blood and platform-dependent batch effects. Additionally, many machine learning models lack transparency and have not been validated in prospective, multi-centre studies, hindering clinical adoption.

Future studies should prioritise large, multi-regional cohorts, harmonised analytical pipelines, and interpretable machine learning models. Integration of transcriptomic and proteomic data, combined with rigorous external validation, will be essential for translating biomarker signatures into clinically viable diagnostic tools.

Current evidence underscores the urgent need for large, multi-centre, and multi-ethnic validation studies, alongside standardised protocols for sample acquisition, data processing, and model evaluation. Prospective longitudinal designs with external cohort replication are essential to demonstrate true predictive value, particularly for progression from LTBI to ATB. Moreover, incorporation of scRNA-seq and advanced ML models (e.g., XGBoost, SVM-RFE) has shown promise in identifying meaningful cellular and metabolic signatures, but requires larger datasets and careful interpretation to ensure biological relevance [t7].

Finally, Discussion of limitations in any biomarker research must acknowledge broader clinical contexts; for example, current WHO target product profiles for TB diagnostics emphasise feasibility in high-burden, low-resource settings, which is seldom addressed in high-complexity omics studies [59].

Technical and biological heterogeneity further complicates biomarker discovery. Differences in sample collection timing, platform technology, and analytical pipelines introduce variability that can obscure true disease-associated signals and hinder reproducibility across studies [21]. In addition, host responses to MTB are dynamic and influenced by a multitude of factors including immune status, co-infection, and genetic background, making universal signatures difficult to define. Transcriptomic differences, for example, reflect diverse immune pathways such as IFN signalling, proliferation, and apoptosis, with considerable overlap between active disease and latent states depending on the cohort and methodology [87].

Many transcriptomic and proteomic studies report promising candidate signatures. For instance, plasma proteomic profiling using label-free approaches identified combinations of host proteins (ACT, AGP1, CDH1) that differentiate pulmonary TB from LTBI with high specificity [turn0search3]. Other large-scale serological screens have identified MTB antigen-specific IgG combinations that show diagnostic potential, although sensitivity and specificity vary widely by cohort and platform [t102]. Complementary transcriptomic work has also identified immune-related expression patterns and signatures with discriminatory power, including potential blood biomarkers for LTBI and ATB distinction [94].

Single biomarkers often reflect isolated components of the host immune response and are therefore vulnerable to biological variability and confounding immune conditions. In contrast, multi-parameter signatures derived using machine-learning algorithms integrate complementary biological signals across multiple pathways, including interferon signalling, innate immune activation, antigen processing, and cellular metabolism. By jointly modelling these features, ML-based classifiers achieve greater robustness, improved signal-to-noise ratios, and superior discrimination between LTBI and ATB. Multiple studies demonstrate that such composite signatures consistently outperform IGRA in sensitivity and specificity, particularly in populations with prior BCG vaccination or immune dysregulation. Nevertheless, despite these encouraging findings, robust external validation of candidate signatures remains scarce. Many studies are constrained by cohort size, geographic homogeneity, or limited representation of key populations such as those with HIV co-infection or extrapulmonary TB [22]. Systematic reviews have underscored that classical immunological assays (e.g., IGRA based on IFN-γ) cannot reliably discriminate LTBI from ATB, and that emerging biomarkers—such as IL-2 responses to latent antigens—may offer improved but not definitive performance [28]. Proteomics research faces parallel challenges. Although host proteins involved in immune regulation, metabolism, and tissue remodelling have been proposed as diagnostic markers across blood, sputum and urine samples, substantial technical obstacles remain. These include the low abundance of clinically relevant proteins, post-translational modifications, and platform dependence that limit standardisation (e.g., differences between mass spectrometry and affinity assays) [13]. Moreover, reliance on single markers appears insufficient; multimarker panels and integrative models often perform better but require rigorous cross-validation and well-powered cohorts [96].

Diagnostic performance was interpreted according to commonly accepted benchmarks: AUC values ≥ 0.90 were considered excellent, 0.80–0.89 good, and 0.70–0.79 moderate. Sensitivity and specificity above 85% were regarded as clinically meaningful for LTBI screening applications. These thresholds align with performance targets proposed for novel TB diagnostics by the World Health Organization and facilitate comparison with established immunological assays. ML models developed to discriminate LTBI from active tuberculosis (ATB) are often evaluated primarily on binary classification accuracy metrics such as sensitivity, specificity, and area under the receiver operating characteristic curve (AUC-ROC). However, false positive and false negative errors represent distinct clinical risks that require explicit consideration, especially in real-world deployment where the distribution of disease and comorbid conditions differs from controlled research cohorts. Transcriptomic feature embeddings generated through dimensionality-reduction and representation-learning techniques preserve biologically meaningful relationships between genes and pathways. These embeddings capture coordinated immune-response patterns rather than isolated gene effects, enabling stratification of LTBI individuals into molecular subtypes characterised by distinct interferon responses, inflammatory profiles, and metabolic states. Such stratification may identify LTBI subgroups with elevated risk of progression to active disease, supporting personalised preventive interventions.

False positive classifications—where individuals without ATB are incorrectly identified as having active disease—can arise when models inadvertently capture non-specific inflammatory signatures rather than M. tuberculosis-specific signals. Transcriptomic and proteomic biomarkers reflecting pathways such as interferon signalling, acute-phase responses, and general immune activation are common to a variety of infectious and inflammatory states. For example:

Elevated interferon-stimulated gene expression (e.g., CXCL10, GBP1, IFITM3) is reported not only in TB but also in viral infections (e.g., influenza, SARS-CoV-2) and granulomatous diseases such as sarcoidosis, leading to potential misclassification in models trained without heterogeneous controls.

Proteomic inflammatory markers (e.g., complement factors, acute-phase proteins) are also elevated in common respiratory diseases and autoimmunity, reducing specificity when used in isolation.

False negative results—where actual cases of ATB are classified as LTBI—can be equally harmful, leading to missed diagnosis, inappropriate management, and ongoing transmission. This can occur due to:

- Heterogeneity of host responses: Individuals with early or paucibacillary TB may not yet exhibit robust or canonical transcriptomic/proteomic signatures, resulting in signal attenuation below model detection thresholds.
- Comorbid conditions: Conditions such as HIV co-infection or immunosuppressive therapy can blunt immune signatures, further reducing model sensitivity if not represented in the training dataset.

These scenarios underscore that relying solely on discriminatory accuracy metrics without understanding the mechanistic drivers of misclassification can mask clinically important error patterns.

Misclassification has different consequences in TB epidemiology and clinical management:

- False positives can lead to unnecessary treatment, psychological burden, health system costs, and potential adverse drug reactions.
- False negatives pose a severe public health risk by enabling continued transmission and delaying appropriate therapy.

Accordingly, future research must incorporate clinically oriented performance metrics beyond AUC-ROC, such as predictive values in defined prevalence settings, decision curve analysis, and cost–benefit modelling.

## 15. Conclusions

In summary, omics-based biomarker discovery combined with AI and ML holds substantial promise for improving the differentiation of LTBI and ATB. However, translation into clinical practice is hindered by cohort limitations, methodological variability, and insufficient validation. Future research must prioritise standardisation, rigorous external validation, and integration of multi-layered data with interpretable ML models to bridge the gap between discovery and routine implementation

This review highlights the substantial potential of omics-informed and ML-enhanced strategies for advancing LTBI diagnostics beyond the limitations of conventional clinical and immunological methods. Despite promising performance metrics, several challenges remain, including the need for multicentre validation, mitigation of batch effects, and improved interpretability of complex ML models. The field is also limited by a predominant focus on cross-sectional diagnosis, underscoring the need for longitudinal cohorts capable of supporting true prognostic modelling [101].

Future progress will depend on the development of harmonised multi-omics datasets, rigorous methodological standards, and accessible point-of-care formats that enable implementation in diverse healthcare settings. The integration of explainable, clinically interpretable ML approaches will be essential to bridge the gap between algorithmic performance and real-world clinical decision-making. As multi-omics integration, deep learning, and diagnostic translation continue to evolve, these technologies have the potential to fundamentally transform LTBI detection, risk stratification, and ultimately, global tuberculosis control.

## Figures and Tables

**Table 1:** Key transcriptomic signatures for the diagnosis of LTBI and active TB.

Study	Data Type	Signature Composition	Performance	Application
Suliman, S. et al., 2016 [24]	RNA-seq, microarray	GBP1, IFITM3, P2RY14, ID3	AUC 0.82–0.89; sensitivity 73–85%; specificity 76–78%	LTBI/ATB differentiation; PoC potential
Vargas, R. et al., 2023 [25]	Whole-blood transcriptomics	GBP2, FCGR1B, SERPIN C1 inhibitor, TUBGCP6, TRMT2A, SDR39U1	AUC 0.93; sensitivity 90.9%; specificity 88.5%	ATB/LTBI differentiation; treatment monitoring
Gong et al., 2021 [12]	GEO RNA datasets + qRT-PCR	UBE2L6, BATF2, SERPING1, VAMP5	Sensitivity ~88%; specificity ~78%; sensitivity 100% (BATF2 + VAMP5)	ATB diagnosis; LTBI vs. ATB; therapy monitoring
Kwan, P.K.W. et al., 2020 [26]	Targeted transcript levels + ML	FCGR1B, GBP1, GBP5	Spec. ~72.7%	LTBI identification vs. uninfected
Qingqing, S. et al., 2024 [27]	GEO whole blood + ML	SLC26A8, ANKRD22, FCGR1B	AUC 0.801	LTBI/ATB discrimination (multi-cohort)

**Table 2:** Diagnostic potential of scRNA-seq for LTBI.

scRNA-seq Task	Description	Diagnostic Relevance
Identification of LTBI-related genes	Differential expression between LTBI, ATB and controls	Molecular signature discovery
Characterisation of immune subpopulations	Profiling T cells, B cells, macrophages, DCs	Detection of LTBI-specific cellular clusters
Assessment of immune activation	IFN-I/II, inflammatory, and metabolic pathways	Identification of high-risk LTBI phenotypes
Predictive modelling	Machine-learning analysis of expression matrices	Early stratification of individuals at risk of progression

**Table 3:** Proteomic biomarkers and machine-learning analysis.

Biomarker Category	Technology	Advantages	Limitations	ML Application
Cytokines/chemokines	Luminex, ELISA	High biological relevance	Wide dynamic range; variability	Boosting, random forest for multiplex panels
Acute-phase proteins	Mass spectrometry	High information content	High cost; pre-analytical complexity	Feature selection, clustering
Complement/innate immunity markers	Targeted proteomics	Pathogenesis relevance	Platform-dependent batch effects	Integration with transcriptomic data

**Table 4:** Neural-network approaches in LTBI diagnostics.

Approach	Data Source	Architecture	Results	Application
CNN for T-SPOT.TB	Immunological imaging	Two-stage CNN + logistic regression	Improved LTBI/ATB differentiation	Enhancement of IGRA interpretation
EfficientNet + MLP-Mixer	Chest radiography	Hybrid deep-learning model	Accuracy 96.3%; sensitivity 95.9%; specificity 96.6%	Screening; disease-activity assessment
Autoencoders	Transcriptomic datasets	Neural representation learning	Extraction of latent molecular subtypes	Predictive modelling
Multimodal transformers	Radiology + omics + immunology	Attention-based transformer	Superior integrated performance	Multimodal LTBI diagnostics

**Table 5:** Limitations of current studies and ML models.

Limitation	Manifestation	Consequences	Required Actions
Insufficient validation	Single-centre cohorts	Poor generalisability	Multi-centre cohorts; TRIPOD compliance
Batch effects	Platform and SOP variability	Spurious signals	Data harmonisation
Low interpretability	Complex ML/DL architectures	Limited clinical acceptance	SHAP/LIME; simplified models
Lack of prognostic evidence	Cross-sectional design	No prediction of progression	Longitudinal cohort studies

## Data Availability

No new data were created or analyzed in this study.

## Abbreviations

AI	Artificial Intelligence
ATB	Active Tuberculosis
AUC	Area Under the Curve
AUC-ROC	Area Under the Receiver Operating Characteristic Curve
BCG	Bacillus Calmette–Guérin
ComBat	Empirical Bayes Method for Batch Effect Correction
COVID-19	Coronavirus Disease 2019
Ct	Cycle Threshold
DCs	Dendritic Cells
DESeq2	Differential Expression Analysis for Sequence Count Data
ELISA	Enzyme-Linked Immunosorbent Assay
ESAT-6	Early Secreted Antigenic Target 6 kDa
FDR	False Discovery Rate
GEO	Gene Expression Omnibus
HCs	Healthy Controls
HC	Healthy Control
IFN-γ	Interferon Gamma
IGRA	Interferon-Gamma Release Assay
LTBI	Latent Tuberculosis Infection
LIME	Local Interpretable Model-Agnostic Explanations
ML	Machine Learning
MIAME	Minimum Information About a Microarray Experiment
MTB	*Mycobacterium tuberculosis*
NTM	Non-Tuberculous Mycobacteria
PBMCs	Peripheral Blood Mononuclear Cells
PCA	Principal Component Analysis
PoC	Point of Care
PPD	Purified Protein Derivative
qRT-PCR/RT-PCR	Quantitative Real-Time Polymerase Chain Reaction
RMA	Robust Multi-array Average
RNA-seq	RNA Sequencing
scRNA-seq	Single-Cell RNA Sequencing
SHAP	SHapley Additive exPlanations
SVM	Support Vector Machine
TB	Tuberculosis
TMM	Trimmed Mean of M-values
TRIPOD	Transparent Reporting of a Multivariable Prediction Model for Individual Prognosis or Diagnosis
UMAP	Uniform Manifold Approximation and Projection
WHO	World Health Organization
